# Increased SUMO-activating enzyme SAE1/UBA2 promotes glycolysis and pathogenic behavior of rheumatoid fibroblast-like synoviocytes

**DOI:** 10.1172/jci.insight.135935

**Published:** 2020-09-17

**Authors:** Cuicui Wang, Youjun Xiao, Minxi Lao, Jingnan Wang, Siqi Xu, Ruiru Li, Xuanxian Xu, Yu Kuang, Maohua Shi, Yaoyao Zou, Qingwen Wang, Liuqin Liang, Song Guo Zheng, Hanshi Xu

**Affiliations:** 1 Department of Rheumatology and Immunology and; 2 Department of Anesthesia, The First Affiliated Hospital, Sun Yat-sen University, Guangzhou, China.; 3 Department of Rheumatism and Immunology, Peking University People’s Hospital, Shenzhen, China.; 4 Division of Rheumatology and Immunology, Department of Internal Medicine, The Ohio State University College of Medicine and The Ohio State University Wexner Medical Center, Columbus, Ohio, USA.

**Keywords:** Immunology, Arthritis, Rheumatology

## Abstract

Fibroblast-like synoviocytes (FLSs) are critical to joint inflammation and destruction in rheumatoid arthritis (RA). Increased glycolysis in RA FLSs contributes to persistent joint damage. SUMOylation, a posttranslational modification of proteins, plays an important role in initiation and development of many diseases. However, the role of small ubiquitin-like modifier–activating (SUMO-activating) enzyme 1 (SAE1)/ubiquitin like modifier activating enzyme 2 (UBA2) in regulating the pathogenic FLS behaviors is unknown. Here, we found an increased expression of SAE1 and UBA2 in FLSs and synovial tissues from patients with RA. SAE1 or UBA2 knockdown by siRNA and treatment with GA, an inhibitor of SAE1/UBA2-mediated SUMOylation, resulted in reduced glycolysis, aggressive phenotype, and inflammation. SAE1/UBA2-mediated SUMOylation of pyruvate kinase M2 (PKM2) promoted its phosphorylation and nuclear translocation and decreased PK activity. Moreover, inhibition of PKM2 phosphorylation increased PK activity and suppressed glycolysis, aggressive phenotype, and inflammation. We further demonstrated that STAT5A mediated SUMOylated PKM2-induced glycolysis and biological behaviors. Interestingly, GA treatment attenuated the severity of arthritis in mice with collagen-induced arthritis and human TNF-α transgenic mice. These findings suggest that an increase in synovial SAE1/UBA2 may contribute to synovial glycolysis and joint inflammation in RA and that targeting SAE1/UBA2 may have therapeutic potential in patients with RA.

## Abstract 2

SUMO-activating enzyme SAE1/UBA2 promotes glycolysis and pathogenic behavior of rheumatoid fibroblast-like synoviocytes through SUMOylation of pyruvate kinase M2.

## Introduction

Rheumatoid arthritis (RA) is an autoimmune-mediated inflammatory disease characterized by progressive synovial inflammation and joint destruction. Fibroblast-like synoviocytes (FLSs), the predominant resident cells in the synovial lining, contribute to both rheumatoid synovial inflammation and joint damage. Constitutively activated FLSs are considered to develop epigenetically imprinted phenotypes that exert aggressive behavior toward cartilage and bone (1, 2). Increasing evidence suggests that targets for prevention of the activation and invasion of FLSs may be promising for ameliorating joint damage in RA (3–5).

In addition to providing energy for physical activity, glucose metabolism is involved in a series of cellular biological processes. Increased glucose metabolism is considered a hallmark of activated and proliferative cells. Targeting glucose metabolism reprogramming may be a promising strategy for new therapeutics of many diseases, including cancer and autoimmune disorders (6–8). It has been shown that glycolytic metabolism is especially enhanced in synovial tissues from patients with RA and contributes to persistent synovial inflammation and joint damage (9–11). Interfering with certain synovial targetable glycolytic enzymes or glycolytic intermediate metabolites may be therapeutic in RA (12). However, the underlying mechanisms that control the glycolytic metabolic phenotype of RA FLSs need to be defined.

SUMOylation, a posttranslational modification of proteins, plays an important role in regulating a number of cellular processes, such as signal transduction and transcription. During SUMOylation, small ubiquitin-like modifier (SUMO) proteins are covalently attached to the target substrates through an enzymatic cascade that is controlled by the sequential action of SUMO-activating enzyme (E1), SUMO-conjugating enzyme (E2), and SUMO ligase (E3) (13). The E1 enzyme is the heterodimer of SUMO-activating enzyme 1 (SAE1)/ubiquitin like modifier activating enzyme 2 (UBA2) that controls critical activation of SUMO proteins. The E2 enzyme Ubc9 then transfers the activated SUMO proteins from UBA2 to a part of the E2 subunit. Finally, E3 ligases, including protein inhibitor of activated STAT (PIAS), Pc2, and RanBp2, transfer SUMO proteins to the lysine residue of the target protein (14, 15). These enzymatic reactions are considered potential targets for small molecules that regulate SUMOylation (14).

The imbalance of SUMOylation and deSUMOylation is associated with the initiation and development of many diseases, including cancers and chronic inflammatory disorders. It has been shown that SUMO/SUMOylation is involved in the pathogenesis of RA (15). For instance, SUMO-1 regulates migration (16) and Fas-induced apoptosis in RA FLSs (17). SUMO-2/3 is involved in the regulation of matrix metalloproteinase 3 (MMP-3) and MMP-13 (18). Knockdown of PIAS3 inhibits aggressive behavior of RA FLSs (19). SUMO specific peptidase 1 induces the accumulation of histone deacetylase 4 on the MMP-1 promoter, inhibiting MMP-1 levels (20). Increased Ubc9 activity may contribute to RA FLS proliferation and invasion (21). However, to date, it is unknown whether the SUMO-activating enzyme SAE1/UBA2 is associated with the pathogenic FLS behavior observed in RA. More interestingly, although a previous study indicated that SUMO-1 modification enhances glycolysis under hypoxia in tumor cells (22), it is still unknown whether SUMOylation is involved in rheumatoid glycolytic metabolism.

The aims of this study were to explore the role of SAE1/UBA2-mediated SUMOylation in regulating glycolysis and pathogenic behavior of RA FLSs and to delineate the underlying mechanisms involved.

## Results

### Increased expression of SAE1/UBA2 in FLSs and synovial tissues from patients with RA.

**Figure 1:**
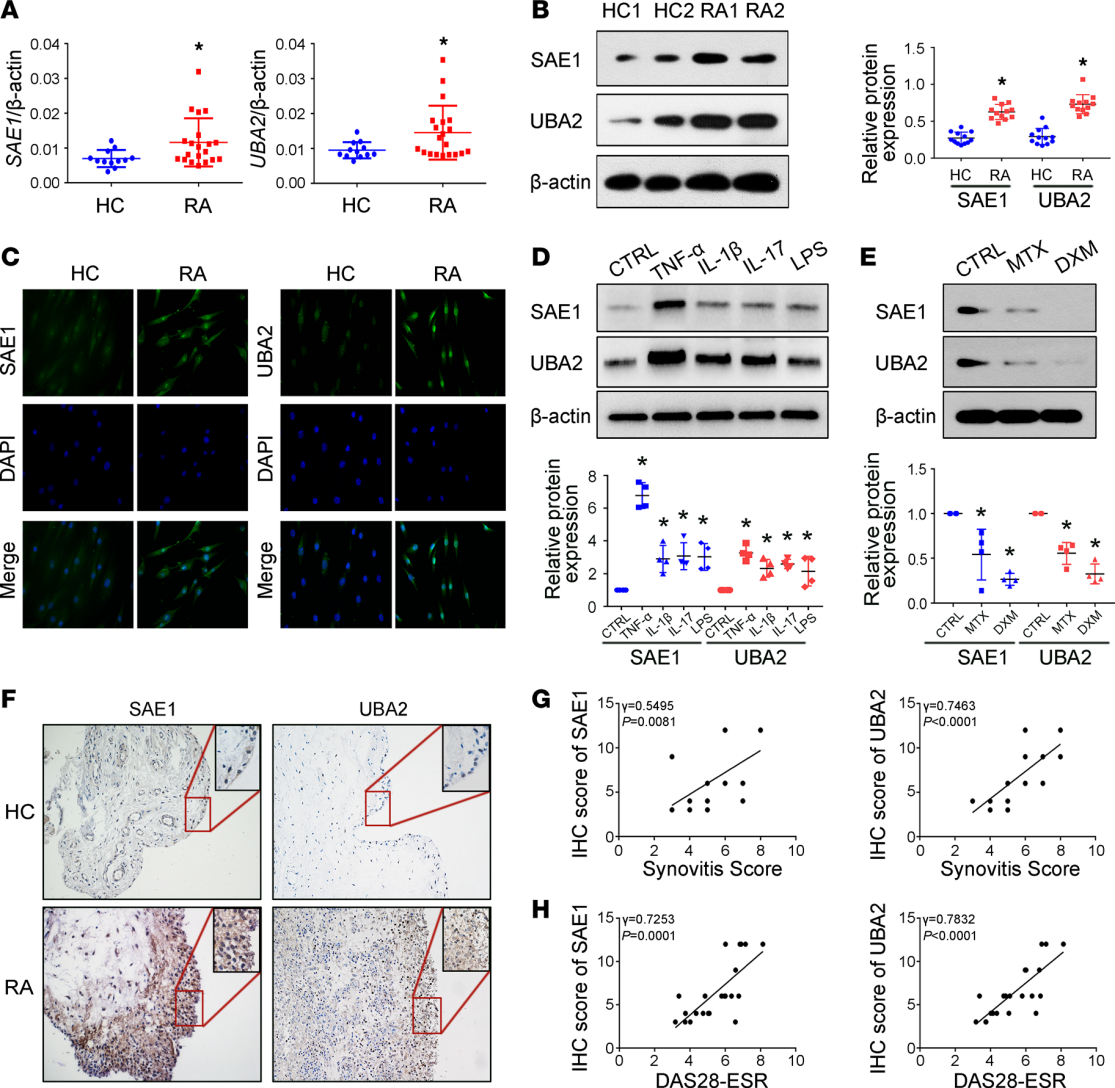
Elevated expression of SAE1 and UBA2 in FLSs and synovial tissues from patients with RA. (**A**) mRNA expression of SAE1 and UBA2 was evaluated as demonstrated by quantitative real-time PCR (RT-qPCR) in FLSs from patients with RA (*n* = 20) and HCs (*n* = 12). (**B**) Protein expression of SAE1 and UBA2 in FLSs was evaluated by Western blot analysis. Data are expressed as the mean ± SD of densitometry quantification (right panel) from 12 RA patients and HC subjects. (**C**) For cellular immunofluorescence (IF) staining, SAE1 or UBA2 (shown in green) and nuclei (shown in blue) were evaluated using fluorescence microscopy, and representative images from 6 RA patients and 6 HC subjects are shown. Original magnification, ×400. (**D**) RA FLSs were treated with TNF-α (10 ng/mL), IL-1β (10 ng/mL), IL-17 (10 ng/mL), or LPS (100 ng/mL) for 24 hours. Data show the mean ± SD of samples from 4 patients with RA. (**E**) RA FLSs were treated with methotrexate (10 μg/mL) or dexamethasone (1 μg/mL) for 24 hours. Data show the mean ± SD of samples from 4 patients with RA. (**F**) Expression of SAE1 and UBA2 assessed by immunohistochemistry in STs from RA patients and HC subjects. The representative images from 22 RA patients and 5 HC subjects are shown. Original magnification, ×200; insets, ×400. (**G** and **H**) Correlation of synovial SAE1/UBA2 protein expression with the synovitis score (**G**) and DAS28-ESR (**H**) in patients with RA. The expression of SAE1 and UBA2 proteins and the synovitis score of 22 patients with RA were evaluated by immunohistochemistry and H&E staining, respectively. Correlation analysis was performed by Spearman’s rank order correlation test. *P < 0.05 versus HC or CTRL, by Student’s *t* test (for **A** and **B**) or 1-way ANOVA (for **D** and **E**).

We found that SAE1 and UBA2 expression was increased in FLSs from patients with RA compared with healthy controls (HCs) (Figure 1, A and B). We also evaluated the subcellular distribution of SAE1 and UBA2 in FLSs by immunofluorescence and found that RA FLSs exhibited markedly enhanced staining for these proteins (Figure 1C). Since proinflammatory cytokines are critical microenvironmental factors in synovial tissue of RA, we determined the effect of these cytokines on the expression of SAE1 and UBA2 in RA FLSs. As shown in Figure 1D, the protein expression of SAE1 and UBA2 was increased in the cells treated with TNF-α, IL-1β, IL-17, or LPS. TNF-α treatment resulted in the most significant increase in SAE1 and UBA2 protein expression; consequently, TNF-α was used as a stimulus for further experiments. We further demonstrated that TNF-α inhibitor treatment reduced SAE1 and UBA2 expression (Supplemental Figure 1; supplemental material available online with this article; https://doi.org/10.1172/jci.insight.135935DS1). We also found that treatment of FLSs with methotrexate or dexamethasone, both of which are important drugs for RA treatment, decreased SAE1 and UBA2 expression (Figure 1E).

SAE1 and UBA2 expression was further evaluated in synovial tissues (STs) by using immunohistochemistry. Compared with that in STs from HCs, SAE1 and UBA2 protein expression was increased in STs from patients with RA and mostly localized in the synovial lining and sublining cells (Figure 1F). We also found positive correlations between synovial SAE1 and UBA2 protein expression and synovitis score as well as Disease Activity Score 28 erythrocyte sedimentation rate (DAS28-ESR) (Figure 1, G and H). Collectively, our findings suggest that increased expression of SAE1 and UBA2 in FLSs may be associated with synovial abnormalities in RA.

In addition, we determined whether T cells and macrophages, the other 2 synovial types of cells, express SAE1 and UBA2 in synovial tissues from patients with RA. We observed that some synovial T cells (CD3^+^) and macrophages (CD68^+^) also expressed SAE1 and UBA2 (Supplemental Figure 2), suggesting that SAE1 and UBA2 might be involved in regulating biological behaviors of rheumatoid synovial T cells and macrophages.

### SAE1/UBA2 inhibition decreases migration, invasion, and proinflammatory cytokine expression in RA FLSs.

**Figure 2:**
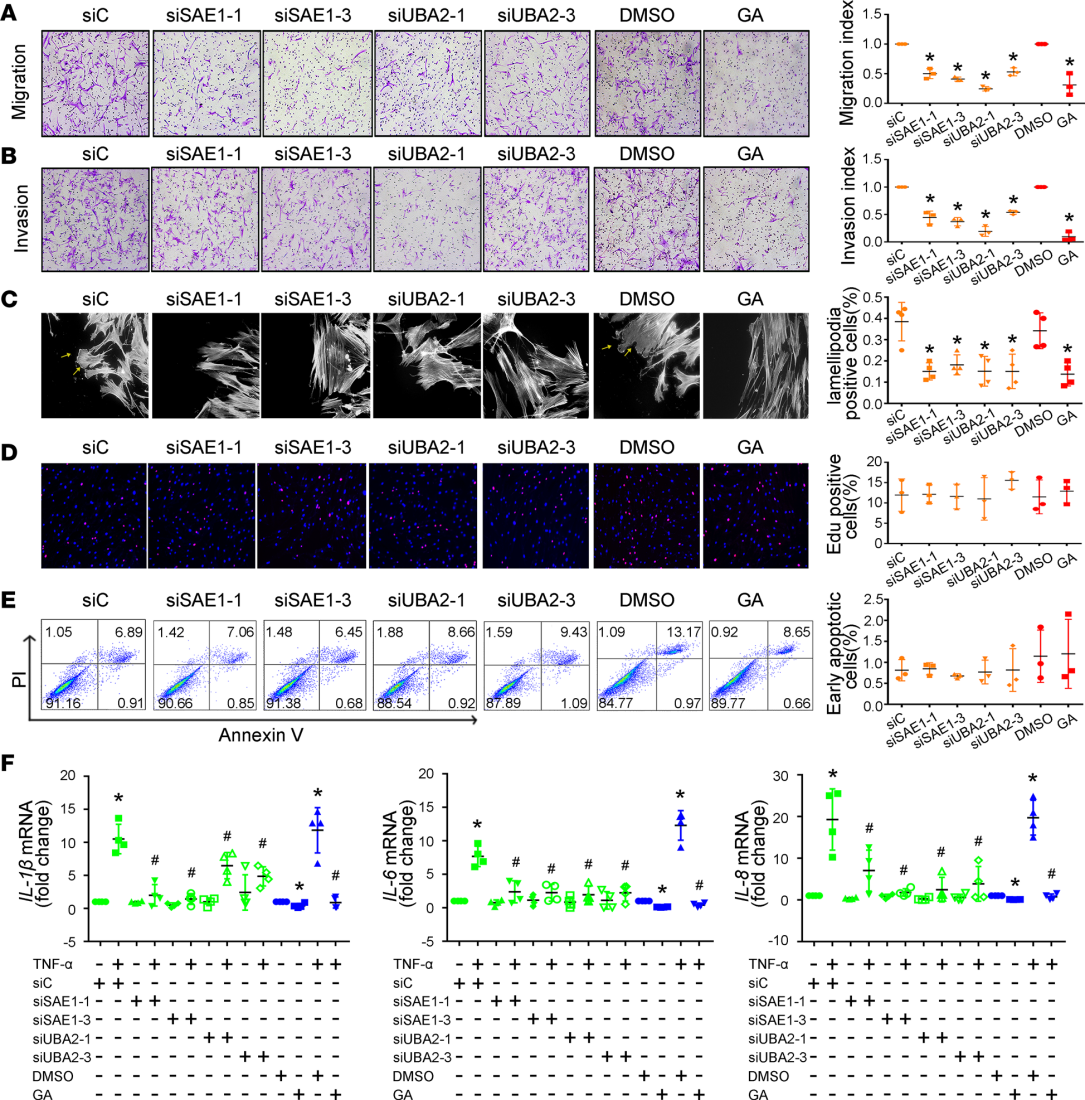
Effects of SAE1/UBA2-mediated SUMOylation inhibition on migration, invasion, and the expression of proinflammatory cytokines in RA FLSs. RA FLSs were transfected with siRNA-1 and siRNA-3 for SAE1 (siSAE1-1, siSAE1-3) or UBA2 (siUBA2-1, siUBA2-3) or control siRNA (siC) or pretreated with ginkgolic acid (GA) (150 μM) for 24 hours. (**A** and **B**) Migration (**A**) and invasion (**B**) were measured with a Boyden chamber. An invasion assay was performed using inserts coated with a Matrigel basement membrane matrix, and 10% FBS was used as a chemoattractant. The migrated or invaded FLSs were stained violet using a Diff-Quik kit (left panel, original magnification, ×100). The migration or invasion index represents the number of migrated cells normalized to that in the siC or DMSO group. Data show the mean ± SD of samples from 3 patients with RA. (**C**) Effect of SAE1/UBA2 inhibition on lamellipodia formation. RA FLSs were plated overnight on coverslips and then fixed and stained with fluorescent phalloidin 6 hours after wounding to visualize polymerized actin in migrating cells. Arrows indicate lamellipodia formation. Graph indicates the number of RA FLSs with positive lamellipodia. (**D**) Effect of SAE1/UBA2 inhibition on proliferation of RA FLSs. An EdU incorporation assay was used to measure cell proliferation. Representative images show proliferation of RA FLSs labeled with EdU (red) and nuclei stained with Hoechst 33342 (blue) (original magnification, ×100). Graphs indicate the mean ± SD of 3 independent experiments involving 3 patients with RA. (**E**) Effect of SAE1/UBA2 inhibition on apoptosis of RA FLSs. The cellular apoptosis rate was evaluated by annexin V and propidium iodide (PI) staining and measured by flow cytometry. Representative flow plots are shown. Mean ± SD percentage of 3 independent experiments involving 3 patients with RA shown. (**F**) Effect of SAE1/UBA2 inhibition on the expression of IL-1β, IL-6, and IL-8. Cytokine expression was evaluated by RT-qPCR. Ct values were normalized to β-actin values. Data are presented as the mean ± SD of 4 independent experiments involving 4 patients with RA. *P < 0.05 versus siC or DMSO; **^#^***P* < 0.05 versus TNF-α+siC or TNF-α+DMSO, by Student’s *t* test (for comparisons between DMSO and GA in panels **A**–**E**) or 1-way ANOVA (for panel **F** and other groups in panels **A**–**E**).

The increased expression of SAE1 and UBA2 in synovial tissues from patients with RA prompted us to investigate the role of these proteins in regulating the biological function of RA FLSs. Interference with siRNA was used to inhibit SAE1 and UBA2 expression. To eliminate nonspecific interference, we designed 3 siRNA oligonucleotide sequences each for SAE1 and UBA2. As shown in Supplemental Figure 3, transfection with all 3 siRNA oligonucleotides decreased SAE1 or UBA2 expression; however, the inhibitory effect of siRNA-1 and siRNA-3 for SAE1 or UBA2 was more prominent. Therefore, siRNA-1 and siRNA-3 for SAE1 or UBA2 were used for subsequent experiments. We determined that transfection with SAE1 or UBA2 siRNA led to a decrease in the migration of RA FLSs compared with control siRNA toward chemoattractant FBS (Figure 2A). We also found a reduction in the migration of RA FLSs treated with ginkgolic acid (GA), a specific inhibitor that reduces SUMOylation by preventing the formation of the SAE1/UBA2-SUMO thioester complex (Figure 2A). Interestingly, transfection with SAE1 or UBA2 siRNA had no effect on the migration of HC FLSs (Supplemental Figure 4A).

The ability to invade cartilage is an important pathogenic behavior of RA FLSs. Thus, we determined the effect of SAE1/UBA2 inhibition on regulating RA FLS invasive behavior by using Matrigel-coated Transwell membranes. We found that transfection with SAE1 or UBA2 siRNA decreased invasion compared with the control siRNA (Figure 2B). GA treatment reduced the invasion of RA FLSs (Figure 2B). SAE1 or UBA2 knockdown also did not influence the invasion of HC FLSs (Supplemental Figure 4B).

Dynamic reorganization of the actin cytoskeleton is critical for cell migration. Herein, we used fluorescent phalloidin staining to visualize polymerized actin in migrating cells shortly after wounding in SAE1/UBA2 suppressed-RA FLSs. As shown in Figure 2C, FLSs transfected with control siRNA or treated with DMSO displayed flat lamellipodia at their leading edge, while cells transfected with SAE1/UBA2 siRNA or treated with GA exhibited reduced lamellipodia formation.

We next evaluated the role of SAE1/UBA2 in the proliferation and apoptosis of RA FLSs. We demonstrated that treatment with GA or transfection with SAE1 or UBA2 siRNA did not affect cell proliferation or apoptosis (Figure 2, D and E). In addition, transfection with SAE1 or UBA2 siRNA did not influence the proliferation or apoptosis of HC FLSs (Supplemental Figure 4, C and D). Our data suggest that SAE1/UBA2 is not involved in the proliferation or apoptosis of RA FLSs and rule out the possibility that the inhibitory effect of SAE1/UBA2 suppression on migration and invasion was related to either proliferation or apoptosis.

Finally, we evaluated the effect of SAE1/UBA2 inhibition on the expression of proinflammatory cytokines by RA FLSs. Transfection with SAE1/UBA2 siRNA or treatment with GA reduced the TNF-α–induced expression of *IL-1β*, *IL-6*, and *IL-8* (Figure 2F). However, SAE1 or UBA2 knockdown did not affect the TNF-α–induced expression of *IL-1β*, *IL-6*, or *IL-8* in HC FLSs (Supplemental Figure 4E).

### Inhibition of SAE1/UBA2-mediated SUMOylation reduces glycolysis in RA FLSs.

**Figure 3:**
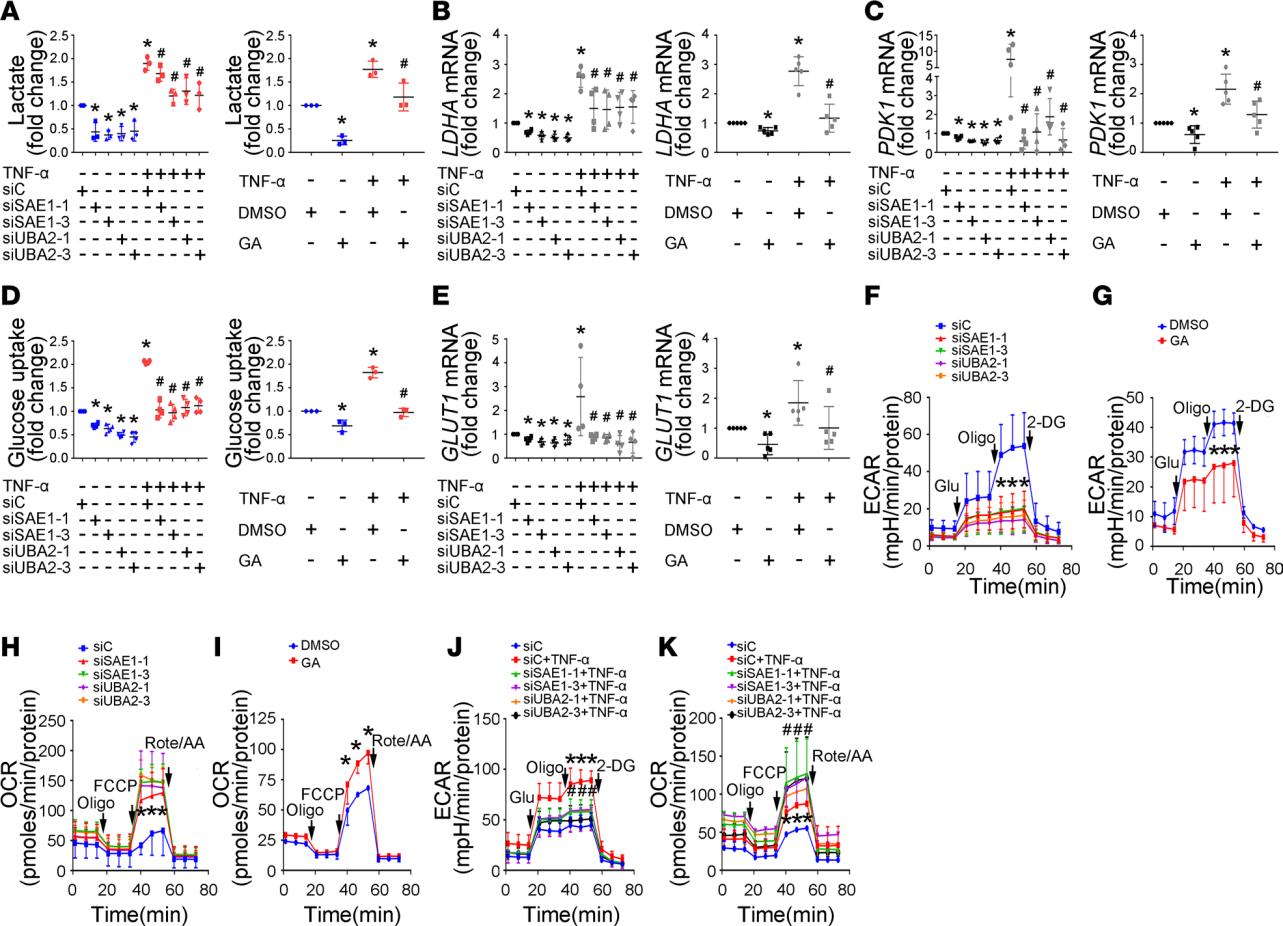
Effect of SAE1/UBA2-mediated SUMOylation inhibition on glycolytic metabolism in RA FLSs. RA FLSs were transfected with siRNA-1 and siRNA-3 for SAE1 (siSAE1-1, siSAE1-3) or UBA2 (siUBA2-1, siUBA2-3) or control siRNA (siC) for 48 hours or pretreated with GA (150 μM) for 24 hours and then stimulated with or without TNF-α (10 ng/mL) for 24 hours. (**A**) Effect of SAE1/UBA2 inhibition on lactate secretion. Lactate levels in the supernatants of cultured RA FLSs were detected using a Lactate Colorimetric Assay Kit. Data show the mean ± SD of samples from 3 patients with RA. (**B** and **C**) Effect of SAE1/UBA2 inhibition on the expression of lactate dehydrogenase A (LDHA) and pyruvate dehydrogenase kinase 1 (PDK1). The expression of LDHA and PDK1 was measured by RT-qPCR. Data show the mean ± SD of samples from at least 4 patients with RA. (**D** and **E**) Effect of SAE1/UBA2 inhibition on glucose uptake and GLUT1 expression. Glucose uptake (**D**) was detected using a colorimetric glucose uptake assay kit. Data show the mean ± SD of samples from at least 3 patients with RA. GLUT1 expression (**E**) was measured by RT-qPCR. Data show the mean ± SD of samples from at least 4 patients with RA. (**F**–**K**) Effect of SAE1/UBA2 knockdown and GA treatment on the extracellular acidification rate (ECAR, **F**, **G**, and **J**) and oxygen consumption rate (OCR, **H**, **I**, and **K**). The data are representative of at least 3 independent experiments (mean ± SD). *P < 0.05 versus siC or DMSO; **^#^***P* < 0.05 versus TNF-α+siC or TNF-α+DMSO, by Student’s *t* test (for **G** and **I**) or 1-way ANOVA (for other panels).

Because it has been reported that SUMO-1 promotes glycolysis in tumor cells (22), we investigated whether SAE1/UBA2-mediated SUMOylation is involved in regulating glycolytic metabolism in RA FLSs. First, we determined the roles of SAE1/UBA2-mediated SUMOylation inhibition in the glycolysis pathway. As shown in Figure 3, A–C, transfection with SAE1 or UBA2 siRNA or treatment with GA reduced the secretion of lactate and the expression of the glycolysis genes *LDHA* and *PDK1*. SAE1/UBA2 knockdown or GA treatment also decreased glucose uptake and glucose transporter 1/solute carrier family family 2 member 1 (*GLUT1*) expression (Figure 3, D and E). Second, we demonstrated that TNF-α treatment increased lactate secretion, glucose uptake, and the expression of *LDHA*, *PDK1*, and *GLUT1*, and these increases were reversed by SAE1/UBA2 knockdown or GA treatment. Third, SAE1/UBA2 knockdown or GA treatment also resulted in a reduced extracellular acidification rate (ECAR), which reflects the overall glycolytic flux, and an elevated oxygen consumption rate (OCR), an indicator of mitochondrial oxidative respiration (Figure 3, F–K). Our findings suggest an important role of SAE1/UBA2-mediated SUMOylation in regulating glycolytic metabolism in RA FLSs.

### SAE1/UBA2 regulates glycolysis and the biological function of RA FLSs through SUMOylation-mediated phosphorylation of PKM2.

**Figure 4:**
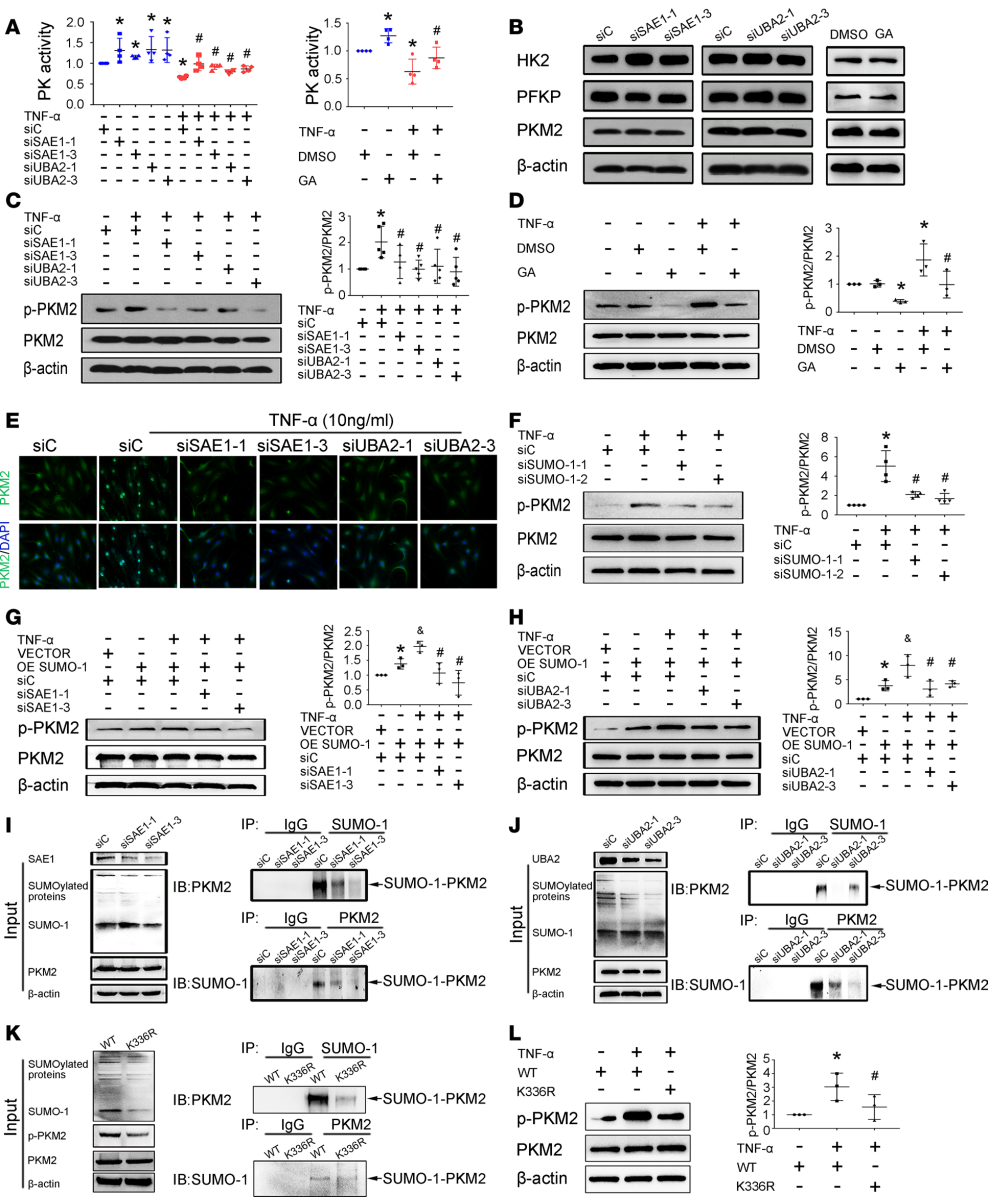
Effect of SAE1/UBA2-mediated SUMOylation inhibition on pyruvate kinase M2 activity in RA FLSs. RA FLSs were transfected with siRNA-1 and siRNA-3 for SAE1 (siSAE1-1, siSAE1-3) or UBA2 (siUBA2-1, siUBA2-3) or control siRNA (siC) for 48 hours or pretreated with GA (150 μM) for 24 hours and then stimulated with or without TNF-α (10 ng/mL) for 24 hours. Protein expression was measured by Western blot. (**A**) Effect of SAE1/UBA2 inhibition on PK activity. Enzyme activity was detected by the indicated kits in the Methods section. Data are expressed as the mean ± SD from 4 independent experiments. (**B**) Effect of SAE1 or UBA2 inhibition on protein expression of HK2, PFKP, and PKM2. (**C** and **D**) Effect of SAE1 or UBA2 knockdown (**C**) or GA treatment (**D**) on TNF-α–induced PKM2 phosphorylation. (**E**) Effect of SAE1 or UBA2 inhibition on TNF-α–induced nuclear translocation of PKM2. For cellular IF staining, PKM2 (green) and nuclei (blue) were evaluated using fluorescence microscopy, and representative images from 3 independent experiments are shown. Original magnification, ×400. (**F**) Effect of SUMO-1 knockdown on TNF-α–induced PKM2 phosphorylation. (**G** and **H**) Effect of SAE1 (**G**) or UBA2 (**H**) knockdown on TNF-α–induced phosphorylation of PKM2 in SUMO-1–overexpressing RA FLSs. (**I** and **J**) Effect of SAE1 or UBA2 knockdown on PKM2 and SUMO-1 interaction. The immunoprecipitates were probed with anti-PKM2 (upper panels) or anti–SUMO-1 (lower panels) antibody. Immunoprecipitations were also performed with IgG as a negative control. Input controls are shown as indicated (left panel). (**K**) Effect of the K336R mutation on PKM2 SUMO-1 modification. MH7A, a human RA FLS cell line, was transfected with or without a PKM2 plasmid carrying a mutation of K^336^ to arginine (K336R). Input controls are shown as indicated (left panel). (**L**) Effect of the K336R mutation on TNF-α–induced PKM2 phosphorylation. Data (**C**, **D**, **F**, **G**, **H**, and **L**) are expressed as the mean ± SD of densitometry quantification of Western blot results from at least 3 independent experiments. *P < 0.05 versus siC or DMSO or VECTOR+siC or WT; **^#^***P* < 0.05 versus TNF-α + siC or TNF-α + DMSO or TNF-α + OE SUMO-1 + siC or TNF-α + WT; ^&^*P* < 0.05 versus OE SUMO-1+ siC, by Student’s *t* test (for comparisons between DMSO and GA in panel **B**) or 1-way ANOVA (for other groups in panel **B** and other panels).

We then sought to gain mechanistic insights into how SAE1/UBA2-mediated SUMOylation may affect glycolytic metabolism. Since the glycolysis pathway is controlled by several key rate-limiting glycolytic enzymes, we evaluated whether SAE1/UBA2 regulates the activity of these enzymes, including hexokinase (HK), phosphofructokinase (PFK), and pyruvate kinase (PK). We found that basic or TNF-α–induced PK activity (Figure 4A), but not HK or PFK activity (Supplemental Figure 5), was increased by SAE1/UBA2 knockdown or GA treatment. However, SAE1/UBA2 knockdown or GA treatment did not affect the protein expression of HK2, platelet-type phosphofructokinase (PFKP), or pyruvate kinase M2 (PKM2) (Figure 4B). These data suggest that SAE1/UBA2-mediated SUMOylation controls glycolytic metabolism by specifically regulating PK activity.

It is well documented that phosphorylation of PKM2 at tyrosine 105 (Y^105^) can induce active tetrameric PKM2 to form an inactive dimer formation by releasing fructose-1, 6-bisphosphate (FBP), resulting in reduction in PK activity and nuclear translocation of PKM2. Therefore, to explore how SAE1/UBA2 modulates PK activity, we investigated the involvement of SAE1/UBA2-mediated SUMOylation in regulating PKM2 phosphorylation. SAE1 or UBA2 knockdown or GA treatment decreased the TNF-α–induced phosphorylation (Figure 4, C and D) and nuclear translocation of PKM2 (Figure 4E and Supplemental Figure 6). Treatment with the PKM2 phosphorylation inhibitor shikonin (SKN) also prevented TNF-α–induced nuclear translocation of PKM2 (Supplemental Figure 7).

We further observed that SUMO-1 knockdown with siRNA reduced the expression of phosphorylated PKM2 (p-PKM2) but not endogenous PKM2 protein (Figure 4F). In contrast, SUMO-1 overexpression increased TNF-α–induced PKM2 phosphorylation, and this increase was reversed by SAE1 or UBA2 knockdown (Figure 4, G and H). SAE1 or UBA2 knockdown with siRNA also reduced protein-conjugated but not free SUMO-1 expression compared with control siRNA (Figure 4, I and J). Furthermore, we demonstrated the interaction of SUMO-1 and PKM2 using coimmunoprecipitation (Supplemental Figure 8). We also found that the SUMO-1/PKM2 interaction was decreased by SAE1 or UBA2 knockdown (Figure 4, I and J) or GA treatment (Supplemental Figure 9). In addition, SUMOsp 2.0 was used to predict potential SUMOylation sites of the PKM2 protein (23). In silico analysis showed that the lysine 336 (K^336^) residue was the most likely to be a SUMO-1 modification site of PKM2. Mutation of K^336^ to arginine substantially blocked PKM2 SUMO-1 modification (Figure 4K) and decreased p-PKM2 expression but did not affect PKM2 expression (Figure 4L), suggesting that K^336^ is the primary SUMOylated site of PKM2 in RA FLSs. Collectively, our data suggest that SAE1/UBA2-mediated SUMOylation of PKM2 promotes its phosphorylation and nuclear translocation in RA FLSs.

Finally, we determined the role of PKM2 in regulating glycolysis and the biological function of RA FLSs. First, we evaluated the expression of p-PKM2 in FLSs and ST from patients with RA. We found increased expression of p-PKM2 in FLSs and ST from patients with RA compared with HCs (Supplemental Figure 10). Second, we designed 3 siRNA oligonucleotide sequences for PKM2 to inhibit p-PKM2 expression. As shown in Supplemental Figure 11, transfection with both siRNA-2 and siRNA-3 decreased the expression of PKM2 and p-PKM2 but not PKM1. Therefore, siRNA-2 and siRNA-3 for PKM2 were used in subsequent experiments. Transfection with PKM2 siRNAs resulted in a decrease in migration; invasion; and the expression of *IL-1β*, *IL-6*, and *IL-8* (Supplemental Figure 12) and a reduction in glucose uptake; lactate secretion; and the expression of *LDHA*, *PDK1*, and *GLUT1* (Supplemental Figure 13). Previous reports indicate that SKN can specifically inhibit PKM2 phosphorylation but not PKM2 protein expression in tumor cells (24). Here, we also found that SKN suppressed TNF-α–induced p-PKM2 but not total PKM2 protein expression in RA FLSs (Supplemental Figure 14). Treatment with SKN reduced migration; invasion; and the expression of *IL-1β*, *IL-6*, and *IL-8* (Supplemental Figure 15), as well as lactate secretion; glucose uptake; and expression of *LDHA*, *PDK1*, and *GLUT1* (Supplemental Figure 16).

### STAT5A is required for SUMOylated PKM2-induced biological functions and glycolysis of RA FLSs.

**Figure 5:**
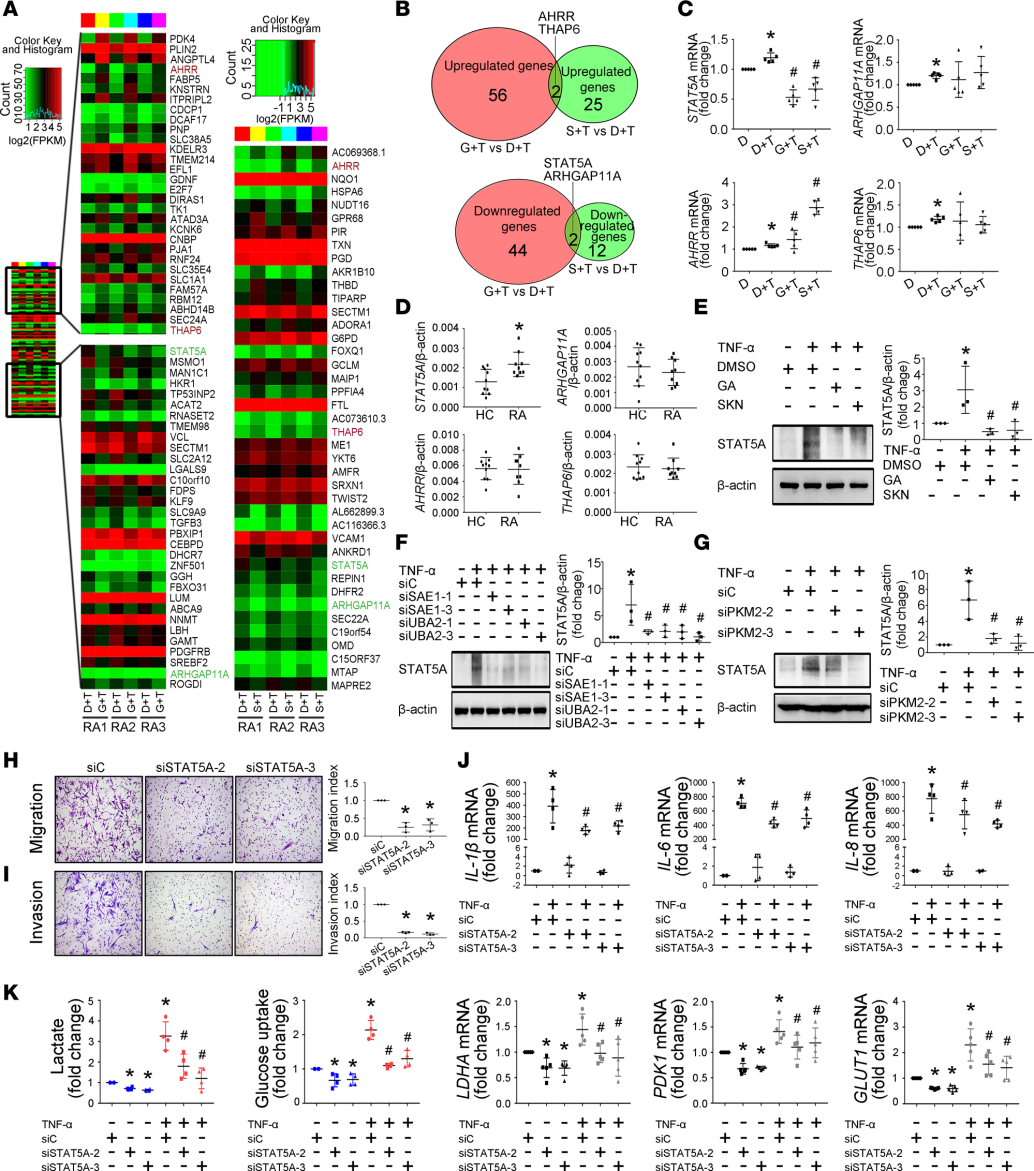
STAT5A mediates SUMOylated PKM2-induced glycolysis and biological functions of RA FLSs. (**A**) Heatmap with hierarchical clustering of differently expressed genes (*P* < 0.05) by RNA sequencing in GA-treated (G+T) or SKN-treated (S+T) versus DMSO-treated (D+T) RA FLSs from 3 patients. (**B**) Venn diagrams showing number of shared and distinct GA- or SKN-treated genes by RNA sequencing in RA FLSs. (**C**) Expression of STAT5A, AHRR, THAP6, and ARHGAP11A was validated by RT-qPCR. Ct values were normalized to β-actin. Data are presented as the mean ± SD of samples from 5 patients with RA. (**D**) Expression of STAT5A, AHRR, THAP6, and ARHGAP11A in RA FLSs and HC FLSs measured by RT-qPCR. Data show the mean ± SD of samples from 9 patients with RA and 10 HCs. (**E**–**G**) Effect of treatment with GA or SKN (**E**), SAE1/UBA2 knockdown, or PKM2 knockdown (**F** and **G**) on TNF-α–induced STAT5A expression measured by Western blot. Data show the mean ± SD of samples from 3 patients with RA. (**H** and **I**) Effect of STAT5A knockdown on migration and invasion of RA FLSs. RA FLSs were transfected with siRNAs for STAT5A (siSTAT5A-2, siSTAT5A-3) or siC. Migration (**H**) and invasion (**I**) were measured with a Boyden chamber. The migrated or invaded FLSs were stained violet using a Diff-Quik kit (left; original magnification, ×100). Data show the mean ± SD of samples from 3 patients with RA. (**J**) Effect of STAT5A knockdown on expression of IL-1β, IL-6, and IL-8. Cytokine expression was measured by RT-qPCR. Data show the mean ± SD of samples from 4 patients with RA. (**K**) Effect of STAT5A knockdown on lactate secretion, glucose uptake, and expression of LDHA, PDK1, and GLUT1. The data represent at least 4 independent experiments (mean ± SD). *P < 0.05 vs. siC or DMSO or HC; ***^#^****P* < 0.05 vs. TNF-α + siC or TNF-α + DMSO, Student’s *t* test (**D**) or 1-way ANOVA (other panels).

To explore how SUMOylated PKM2 regulates biological functions and glycolysis, we evaluated the transcriptome of GA- or SKN-treated RA FLSs compared with untreated control using RNA sequencing analysis. The data were analyzed for differential expression with a nominal *P* < 0.05. GA treatment induced downregulation of 46 genes and upregulation of 58 genes compared with DMSO treatment. SKN treatment resulted in downregulation of 14 genes and upregulation of 27 genes compared with DMSO treatment (Figure 5A). Figure 5B shows a Venn diagram of the overlap of the significantly expressed genes between GA and SKN treatment. We found that in the overlap of both GA and SKN treatment, *STAT5A* and Rho GTPase activating protein 11A (*ARHGAP11A*) were significantly downregulated, and *THAP6* and aryl-hydrocarbon receptor repressor (*AHRR*) were significantly upregulated. RT-qPCR analysis confirmed the significant changes in the *STAT5A* and *AHRR* genes but not in *THAP6* or *ARHGAP11A* (Figure 5C). We further found that only *STAT5A* expression was increased in RA FLSs compared with HC FLSs (Figure 5D). Treatment with GA or SKN or SAE1/UBA2 knockdown also decreased STAT5A protein expression (Figure 5, E–G). We further determined that the K336R mutant of PKM2 reduced STAT5A expression (Supplemental Figure 17). These data indicate that STAT5A may mediate SUMOylated PKM2-induced biological functions and glycolysis of RA FLSs.

Next, we observed the role of STAT5A in regulating RA FLS biological behaviors and glycolysis. We designed 3 siRNA oligonucleotide sequences to inhibit the expression of STAT5A. As shown in Supplemental Figure 18, transfection with both siRNA-2 and siRNA-3 decreased STAT5A expression. Therefore, siRNA-2 and siRNA-3 for STAT5A were used in subsequent experiments. We demonstrated that STAT5A siRNA transfection decreased the migration and invasion of RA FLSs (Figure 5, H and I) and reduced TNF-α–induced expression of *IL-1β*, *IL-6*, and *IL-8* compared with control transfection (Figure 5J). STAT5A knockdown also resulted in downregulation of glucose uptake, lactate secretion, and the expression of *LDHA*, *PDK1*, and *GLUT1* (Figure 5K). These results suggest the involvement of STAT5A in regulating the biological function and glycolysis of RA FLSs and that STAT5A might be a novel target for RA treatment.

### Treatment with GA attenuates the severity of arthritis in mice with collagen-induced arthritis.

**Figure 6:**
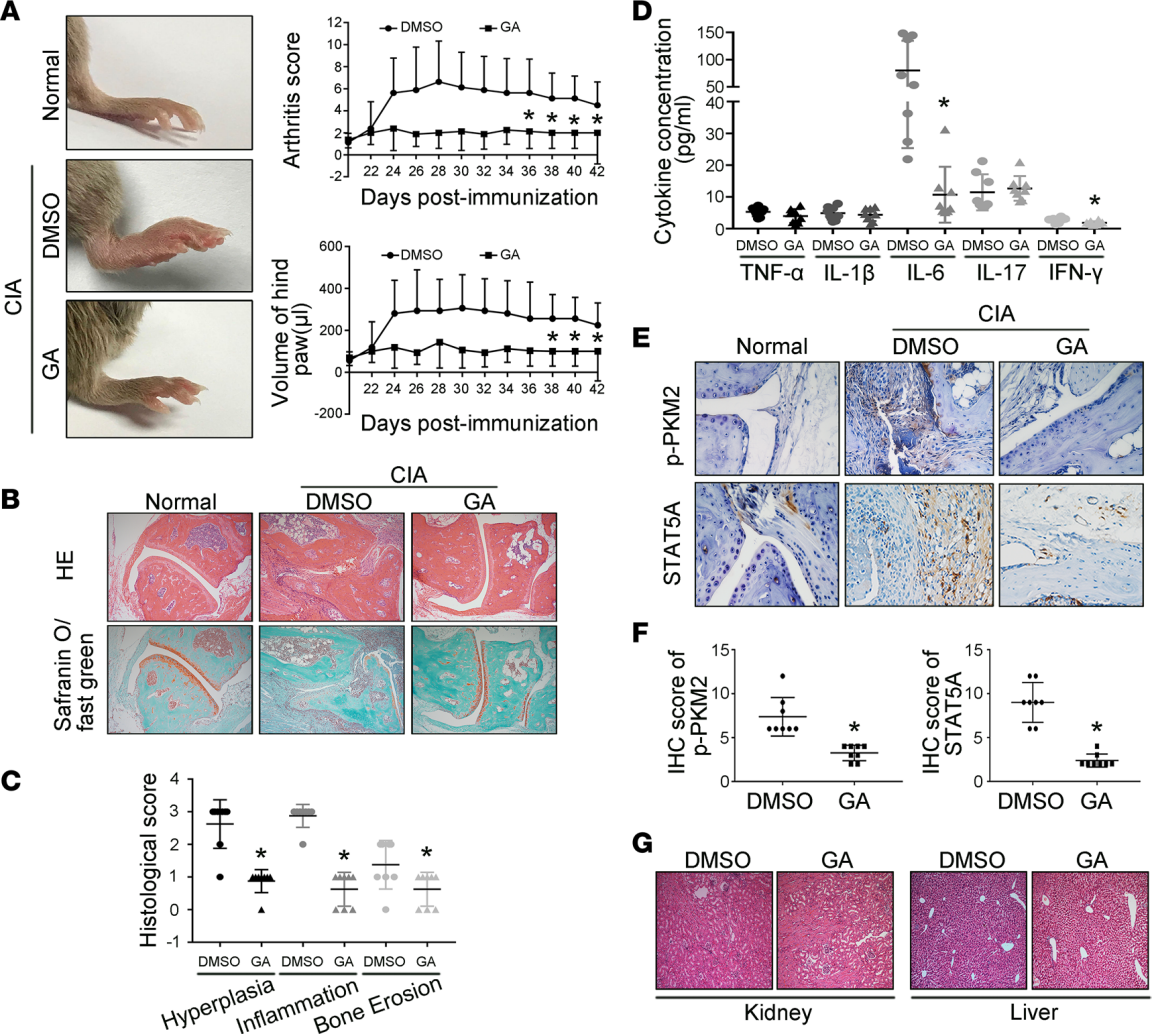
Effect of the SAE1/UBA2 inhibitor GA on the severity of arthritis in mice with CIA. DBA/1J male mice were immunized with bovine type II collagen in complete Freund’s adjuvant and administered a booster injection 21 days later to induce CIA. On the 22nd day, the mice were injected i.p. with GA (30 mg/kg/d) or DMSO (as a model control) daily for 21 days. (**A**) Effect of GA on clinical scores and paw swelling (changes in volume) in CIA mice. The values in **A** are the mean ± SD in 8 mice treated with GA or 8 mice treated with DMSO. (**B** and **C**) Histological appearance of the joints of normal control and CIA mice treated with DMSO (*n* = 8) or GA (*n* = 8). H&E staining was used to evaluate synovial infiltration, cartilage erosion, and bone loss, whereas the lower panel shows safranin O/fast green staining demonstrating proteoglycan depletion (**B**). Original magnification, ×100. The scores for synovial inflammation, cartilage erosion, proteoglycan depletion, and bone loss are shown as the mean ± SD in **C**. (**D**) Effect of GA treatment on levels of cytokines in ankle tissues of CIA mice. The concentration of cytokines was measured with the Milliplex Map Mouse Cytokine Kit. (**E** and **F**) Expression of p-PKM2 and STAT5A, measured by immunohistochemical staining, in synovial tissue from CIA mice. Representative images (**E**) and quantification of the percentage of p-PKM2–positive and STAT5A-positive cells (**F**) of normal mice (*n* = 5), DMSO-treated mice (*n* = 8), and GA-treated (*n* = 8) mice (original magnification, ×400). (**G**) Effect of GA on the kidney and liver of mice with CIA. Photomicrographs show the histopathology of the kidney and liver in mice treated with GA or DMSO. Original magnification, ×100. *P < 0.05 versus DMSO, Student’s *t* test (for **C**, **D**, and **F**) or 2-way ANOVA (for **A**).

The in vivo effect of SAE1/UBA2-mediated SUMOylation by GA on joint inflammation in RA was evaluated first in mice with collagen-induced arthritis (CIA). As shown in Figure 6A, i.p. injection of GA reduced clinical scores and paw swelling (change in volume) compared with DMSO treatment. GA administration also attenuated the inflammatory cell infiltration, synovial hyperplasia, and the cartilage and bone destruction of the joint (Figure 6, B and C). We also found that GA treatment reduced levels of IL-6 and IFN-γ, but not TNF-α, IL-1β, and IL-17, in ankle tissues of CIA mice (Figure 6D). We further determined that synovial expression of p-PKM2 and STAT5A was decreased in GA-treated CIA mice compared with DMSO-treated mice (Figure 6, E and F). GA treatment also reduced the frequency of joint fibroblasts, macrophages, and T cells (Supplemental Figure 19). In addition, we found that synovial macrophages and T cells also expressed SAE1 and UBA2 in CIA mice, and the percentages of those macrophages and T cells were reduced in GA-treated CIA mice compared with DMSO-treated mice (Supplemental Figure 20).

Body weight was not significantly different between the GA and DMSO groups during the course of the experiment (Supplemental Table 3). We also observed no significant alterations in serum glucose levels, liver (alanine aminotransferase [ALT] and aspartate aminotransferase [AST] levels) parameters, and renal (serum creatinine levels) parameters in mice treated with GA (Supplemental Table 3). We further found no significant histopathological changes in kidneys and livers removed from GA-treated mice compared with those of DMSO-treated mice (Figure 6G). These data suggest the safety of GA treatment in CIA mice.

### Administration of GA attenuates the severity of arthritis in human TNF-α transgenic mice.

**Figure 7:**
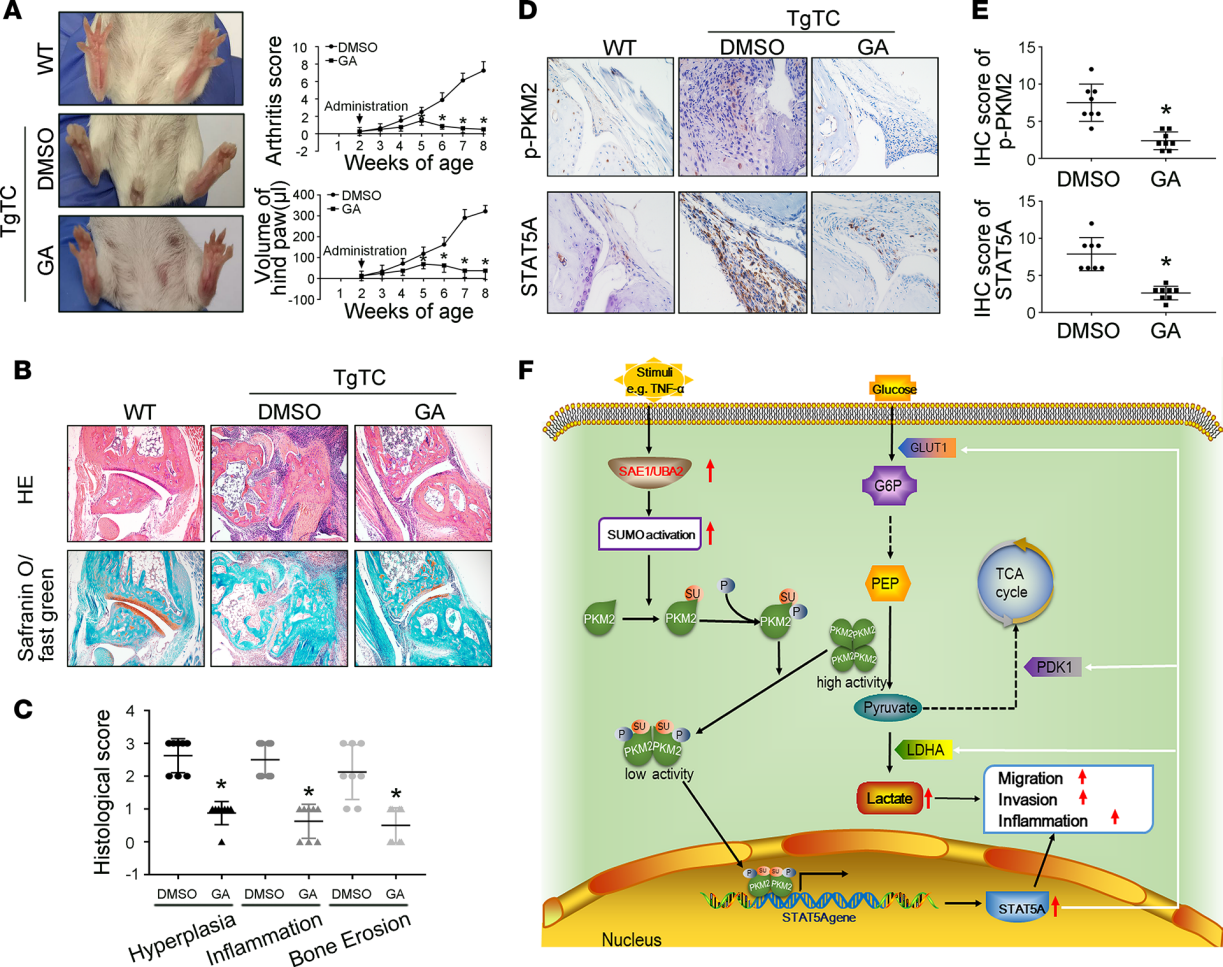
Effect of the SAE1/UBA2 inhibitor GA on the severity of arthritis human TgTC mice. A total of 16 female 2-week-old TgTC mice were grouped randomly to be administered an i.p. injection of GA (30 mg × kg^–1^, every day, *n* = 8) or DMSO (vehicle, *n* = 8) for 6 weeks. (**A**) Effect of GA on clinical scores and paw swelling (changes in volume) in TgTC mice. The values are the mean ± SD in 8 mice treated with GA or 8 mice treated with DMSO. (**B** and **C**) Histological appearance of the joints of normal control and TgTC mice treated with DMSO (*n* = 8) or GA (*n* = 8). H&E staining was used to evaluate synovial infiltration, cartilage erosion, and bone loss, whereas the lower panel shows safranin O/fast green staining demonstrating proteoglycan depletion (**B**). Original magnification, ×100. The scores for synovial inflammation, cartilage erosion, proteoglycan depletion, and bone loss are shown as the mean ± SD in **C**. (**D** and **E**) p-PKM2 and STAT5A expression, measured by immunohistochemical staining, in synovial tissue from TgTC mice. Representative images (**D**) of p-PKM2 and STAT5A expression from normal mice (*n* = 5), DMSO-treated mice (*n* = 8), and GA-treated (*n* = 8) mice (original magnification, ×400). Quantification of the percentage of p-PKM2–positive and STAT5A-positive cells of DMSO-treated and GA-treated mice are shown as the mean ± SD in **E**. (**F**) Diagram of the proposed role of SAE1/UBA2 in regulating glycolytic metabolism and biological behaviors of RA FLSs. *P < 0.05 versus DMSO, by Student’s *t* test (for panels **C** and **E**) or 2-way ANOVA (for panel **A**).

Since SAE1/UBA2 function is predominately dependent on TNF-α–induced activation in vitro, we also determined the effect of GA on joint inflammation in TgTC mice, a human TNF-α transgenic model of arthritis. GA administration reduced clinical scores compared with DMSO treatment (Figure 7A). GA treatment also attenuated the synovial inflammation, hyperplasia, and the cartilage and bone destruction of the joint (Figure 7, B and C). We further demonstrated that synovial expression of p-PKM2 and STAT5A was decreased in GA-treated TgTC mice compared with DMSO-treated mice (Figure 7, D and E).

## Discussion

In this study, we showed the increased expression of SAE1 and UBA2 in FLSs and STs from patients with RA. SAE1/UBA2 knockdown or GA treatment also decreased glycolysis, aggressive behavior, and the inflammatory response in RA FLSs. Furthermore, our studies revealed that the SAE1/UBA2-mediated SUMOylation of PKM2 promoted its phosphorylation and nuclear translocation, and STAT5A mediates the SUMOylated PKM2-induced biological behaviors and glycolysis of RA FLSs. Interestingly, GA treatment attenuated the severity of arthritis in CIA and TgTC mice. Our data suggest that increased SAE1/UBA2-mediated SUMOylation may contribute to abnormal glycolysis and pathogenic behaviors of RA FLSs by targeting PKM2-mediated STAT5A.

Recent studies indicate that SAE1/UBA2, a heterodimer that catalyzes the first step of the SUMOylation cascade, is involved in regulating tumor growth and metastasis. For instance, high SAE1 and UBA2 (SAE2) levels are correlated with decreased survival and increased metastatic ability in patients with breast cancer (25). Hepatocellular carcinoma patients with elevated SUMO E1 expression have poor survival (26). SAE1 knockdown results in synthetic lethality in high Myc–expressing B cell lymphoma (27). In this study, we demonstrated increased expression of SAE1/UBA2 in synovial tissues from patients with RA, which was associated with the severity of synovitis in patients with RA. SAE1/UBA2-mediated SUMOylation inhibition decreased the migration, invasion, and expression of proinflammatory cytokines in RA FLSs. Administration of GA improved the severity of arthritis in CIA mice. These data suggest that increased synovial SAE1/UBA2 contributes to FLS-mediated synovial aggressive behavior and inflammation in RA.

Increasing evidence shows the important roles of glycolytic metabolism in regulating behaviors of RA FLSs (5, 28); however, the underlying mechanisms that control RA FLS glycolysis are still ill-defined. A previous study showed that SUMO-1 modification contributed to hypoxia-induced enhanced glycolysis in tumor cells (20). In this study, we determined that SAE1/UBA2 knockdown or GA treatment decreased glycolytic metabolism by specifically increasing PK activity but not PKM2 protein expression. It is well known that PKM2 phosphorylation at Y^105^ can induce active tetrameric PKM2 to inactive dimer formation, therefore leading to a decrease in PK activity (29–31). We revealed that inhibition of SAE1/UBA2-mediated SUMOylation blocked TNF-α–induced PKM2 phosphorylation at Y^105^, and treatment with SKN, an inhibitor of PKM2 phosphorylation, suppressed the abnormal glycolytic metabolism of RA FLSs. These data provide evidence that increased SAE1/UBA2-mediated SUMOylation downregulates PK activity by promoting PKM2 phosphorylation in RA FLSs. Indeed, consistent with our findings, SUMO overexpression also increased endogenous p38 phosphorylation in response to *Helicobacter pylori* infection in the gastric epithelial cell line AGS (32).

Previous studies show that PKM2 phosphorylation at Y^105^ promotes active tetrameric PKM2 into an inactive dimer formation by releasing FBP, leading to increased nuclear translocation of PKM2 (29, 30). In addition, ERK1/2-dependent phosphorylation of PKM2 is associated with nuclear translocation of PKM2 (33). In contrast, forcing dimeric PKM2 into a tetramer conformation with ML265 blocks its nuclear translocation induced by LPS (34). In our work, we found that TNF-α–induced nuclear translocation of PKM2 was prevented by SAE1/UBA2 knockdown and GA treatment, suggesting that SAE1/UBA2-mediated SUMOylation promotes the phosphorylation and nuclear translocation of PKM2 in RA FLSs. Similar to our findings, recent studies indicate that posttranslational modification of PKM2 through SUMOylation by PIAS3 and acetylation by p300 acetyltransferase can block binding of PKM2 to FBP and promote its nuclear translocation (35, 36).

PKM2 is a critical enzyme that catalyzes the last step of glycolysis. Tetrameric PKM2 is more active than dimeric PKM2, and phosphorylation of PKM2 at Y^105^, which promotes the tetramer-to-dimer conversion of PKM2, leads to an increased accumulation of glycolytic intermediates (37). We showed that blockade of SAE1/UBA2-mediated SUMOylation as well as inhibition of PKM2 phosphorylation reduced TNF-α–induced lactate secretion, glucose uptake, and expression of the glycolysis genes *LDHA* and *GLUT1*. PDK is a key enzyme that decreases pyruvate entry into the tricarboxylic acid cycle, thereby increasing the secretion of lactate. Here, we showed the inhibitory effect of SAE1/UBA2 knockdown or a PKM2 phosphorylation inhibitor on *PDK1* expression and lactate levels, further supporting the notion that SAE1/UBA2-mediated SUMOylation modulates glycolysis by targeting PKM2 phosphorylation. Because previous reports have shown that lactate promotes migration, invasion, and inflammatory responses in RA FLSs (8, 10), our findings indicate that the aberrant biological behaviors of RA FLSs induced by SAE1/UBA2-mediated SUMOylation may be partly due to the accumulation of lactate resulting from increased synovial glycolysis.

In addition to its well-known involvement in glycolysis, PKM2 has nonmetabolic functions. Nuclear PKM2 can promote cytokinesis and cell cycle progression and epigenetically regulate gene transcription in tumor cells (38–40). PKM2 regulates the release of high mobility group box-1, a nuclear protein that acts as a potent proinflammatory cytokine, by activated macrophages through interaction with HIF-1α (41). Nuclear translocation of dimeric PKM2 boosts the transcription of *IL-1β* and *IL-6* in LPS-activated macrophages from patients with coronary artery disease (42). Consistent with these studies, we determined that PKM2 knockdown or SKN treatment suppressed migration, invasion, and the expression of *IL-1β*, *IL-6*, and *IL-8* by TNF-α–treated RA FLSs.

To further explore how SUMOylation-mediated PKM2 phosphorylation regulates RA FLS biological behaviors and glycolysis, we used RNA sequencing analysis to identify relevant downstream targets. We found that inhibition of SAE1/UBA2-mediated SUMOylation or PKM2 phosphorylation decreased the gene and protein expression of STAT5A, an important STAT family transcription factor, indicating that PKM2 might be a new regulator of STAT5A. Indeed, consistent with our findings, it has been shown that PKM2 can interact with several transcription factors, such as STAT3, HIF-1α, and histone H3 (40, 43, 44). However, it would be interesting to further investigate how PKM2 regulates STAT5A gene expression.

Emerging evidence indicates the involvement of STAT5 in immunity and inflammatory responses. STAT5 plays a role in IL-2 signal–mediated proliferation and differentiation of T cells (45, 46). STAT5A/5B-deficient mice show loss of tolerance and autoimmunity in multiple organs (47). Herein, we demonstrated that STAT5A expression was increased in RA FLSs compared with HC FLSs and that STAT5A knockdown decreased the expression of *IL-1β*, *IL-6*, and *IL-8*. In addition, activated STAT5 can promote the growth and invasion of cancer cells (48). We also showed that STAT5A knockdown reduced the migration and invasion of RA FLSs. Interestingly, we found for what is likely the first time that STAT5A knockdown reduced lactate secretion, glucose uptake, and the expression of proglycolytic genes, suggesting the involvement of STAT5A in glycolytic metabolism in RA FLSs. Collectively, our data suggest an important role for STAT5A in regulating RA FLS-mediated rheumatoid synovial aggression and inflammation.

In summary, our data show that increased SAE1/UBA2 promotes SUMOylation and sequential phosphorylation of PKM2, resulting in a reduction in PK activity, promotion of the nuclear translocation of PKM2, and an increase in STAT5A expression, thereby shunting glucose from oxidative phosphorylation metabolism to glycolysis and promoting aggressive and inflammatory actions in RA FLSs (Figure 7F). Our study reveals an important link between SAE1/UBA2-mediated SUMOylation and glycolytic metabolism alterations in synovial aggression and inflammation, which characterize joint abnormalities in RA.

## Methods

### Reagents and antibodies.

Recombinant human TNF-α, IL-1β, and IL-17α were purchased from R&D Systems (Bio-Techne), and LPS was obtained from MilliporeSigma. DMEM, FBS, antibiotics, trypsin EDTA, PBS, and other products for cell culture were purchased from Invitrogen, Thermo Fisher Scientific. The primary antibodies used were as follows: anti-SAE1 (ab38434, Abcam), anti-UBA2 (ab189289, Abcam), anti–SUMO-1 (ab11672, Abcam), and anti-STAT5A (ab32043, Abcam) were purchased from Abcam. Anti-HK2 (2867, Cell Signaling Technology), anti-PFKP (8164, Cell Signaling Technology), anti-PKM2 (4053, Cell Signaling Technology), and anti–p-PKM2 (3827, Cell Signaling Technology) were purchased from Cell Signaling Technology. Anti–β-actin (A5441) was purchased from MilliporeSigma. GA (15:1) was purchased from MilliporeSigma. SKN was purchased from Selleckchem.

### Preparation of human STs and FLSs.

STs were obtained from patients with active RA (20 women and 4 men, aged 54.8 ± 7.5 years) who were undergoing synovectomy of the knee joint at The First Affiliated Hospital, Sun Yat-sen University. RA was diagnosed according to the 2010 revised criteria of the American College of Rheumatology/European League Against Rheumatism classification criteria (49). RA patient demographics are provided in Supplemental Table 1. STs of HCs were obtained from 12 patients who underwent a traumatic single above-knee amputation and had no history of acute or chronic arthritis.

STs were cut into small pieces, placed on a cell culture dish for 6 hours for cell attachment, and then incubated with fresh complete medium. The cells were cultured in DMEM with 10% FBS at 37°C and 5% CO_2_. In our study, FLSs were used from passages 4–6, during which time they were a homogeneous population of cells (<1% CD11b positive, <1% phagocytic, and <1% FcgRII and FcgRIII receptor positive). In addition, human RA FLS cell line MH7A (Jennio Biotech Co.,Ltd), cultured as primary FLSs, was used in some experiments.

### Quantitative real-time PCR.

Total RNA was prepared by the TaKaRa PrimeScript RT Reagent Kit according to the manufacturer’s protocol. RT-qPCR was performed using the Bio-Rad CFX96 system. All experiments were carried out in triplicate. The primers used for RT-qPCR are listed in Supplemental Table 2. To quantify the relative expression of each gene, Ct values were normalized to the endogenous reference values (ΔCt = Ct target – Ct β-actin) and compared with a calibrator using the ΔΔCt method (ΔΔCt = ΔCt sample – ΔCt calibrator).

### Western blot analysis.

Cells were lysed using radioimmunoprecipitation assay lysis buffer (50 mM Tris-HCl, pH 7.4, 150 mM NaCl, 1% NP-40, and complete protease inhibitor cocktail) for 15 minutes on ice and then centrifuged at 12,000*g* for 15 minutes at 4°C. The bicinchoninic acid protein assay (Pierce, Thermo Fisher Scientific) was used to detect protein concentrations. Equal amounts of protein were solubilized in Laemmli buffer (62.5 mM Tris-HCl pH 6.8, 10% glycerol, 2% SDS, 5% β-mercaptoethanol, and 0.00625% bromophenol blue), boiled for 5 minutes, and then separated by SDS-PAGE and transferred to polyvinylidene diﬂuoride membranes. The membranes were probed with primary antibodies as indicated in Tris-buffered saline–Tween containing 5% nonfat milk at 4°C overnight and then incubated with the appropriate secondary antibodies (anti–rabbit IgG, 7074, Cell Signaling Technology; and anti–mouse IgG, 7076, Cell Signaling Technology) for 1 hour at room temperature. Immunoreactive bands were visualized using enhanced chemiluminescence (GE Healthcare). Each blot is representative of at least 3 similar independent experiments.

### Transfection of siRNAs.

We obtained SAE1, UBA2, PKM2, SUMO-1, and STAT5A siRNAs and control siRNA from RiboBio. The target sequences of the siRNAs are listed in Supplemental Table 3. FLSs were seeded in 6-well plates at 60%–70% confluence and transiently transfected with the above siRNAs (100 nM) or the corresponding control siRNA using Lipofectamine 3000 (Invitrogen, Thermo Fisher Scientific). Experiments were performed 48 hours after transfection.

### Infection of overexpression lentivirus.

The SUMO-1 overexpression lentivirus was obtained from GeneChem, in which the SUMO-1 gene (NM_003352) or scramble sequence was subcloned into the GV365 vector (GeneChem). Lentiviruses were produced by cotransfection of HEK293T cells with the expression vectors and GFP-labeled helper plasmids using Lipofectamine 3000 according to the manufacturer’s protocol. Lentiviral particles were harvested from cell supernatants 48 and 72 hours after transfection. For infection, FLSs were treated with the lentiviruses in the presence of polybrene (10 mg/mL, Santa Cruz Biotechnology) for 6–8 hours at 37°C. Afterward, the virus-containing medium was replaced with fresh medium. The infection efficiency of SUMO-1 overexpression lentiviruses was examined using RT-qPCR and Western blot analysis.

### Transfection of plasmids.

To determine the SUMOylation sites of the PKM2 protein, we used SUMOsp 2.0 (23) to predict the potential SUMOylation sites of PKM2. In silico analysis suggested that the K^336^ residue was the most likely site of PKM2 modification by SUMO-1. Therefore, we constructed PKM2 plasmids, including PKM2 WT or mutant type, which contained a mutation from K^336^ to arginine (K336R) (Sangon Biotech). Human RA FLS cell line MH7A (Jennio Biotech Co.,Ltd) was seeded in 6-well plates at 60% to 70% confluence and transiently transfected with plasmids using Lipofectamine 3000. Experiments were performed 48 hours after transfection. All assays were conducted in triplicate.

### Detection of cell migration and invasion.

FLS migration in vitro was performed by the Boyden chamber method using a filter of 6.5 mm diameter and 8.0 μm pore size (Transwell, Corning Inc.). Briefly, DMEM containing 10% FBS as a chemoattractant was placed in the lower wells, and cells suspended in serum-free DMEM at a final concentration of 2 × 10^4^ cells/mL were placed in the upper wells. The chamber was incubated at 37°C under 5% CO_2_ for 6 hours, and then the nonmigrating cells were removed from the upper surface of the filter using a cotton swab. The filters were fixed in methanol for 15 minutes and stained with 0.1% crystal violet (MilliporeSigma) for 15 minutes. Chemotaxis was quantified using an optical microscope (Olympus BX63) to count the stained cells that had migrated to the lower side of the filter. The stained cells were counted as the mean number of cells per 10 random fields for each assay. For the detection of invasion in vitro, similar experiments were performed using inserts coated with a Matrigel basement membrane matrix (BD Biosciences) and DMEM containing 15% FBS as a chemoattractant.

### Immunohistochemistry.

For immunohistochemistry, ST sections were deparaffinized, followed by incubation with 5% serum in PBS for 2 hours to block nonspecific binding and then incubation with 3% H_2_O_2_ for 10 minutes to block endogenous peroxidase activity. The expression of SAE1 and UBA2 was determined by staining with polyclonal rabbit anti-human SAE1 and UBA2 antibodies overnight at 4°C. Polyclonal goat anti-rabbit antibodies (PV-9003, Zhongshan Jinqiao Biotechnology) labeled with horseradish peroxidase were used as secondary antibodies for 1 hour at room temperature. The results were revealed using diaminobenzidine.

### IF staining.

RA FLSs growing on glass coverslips were fixed with 4% paraformaldehyde for 15 minutes and then permeated with 0.1% Triton X-100 in PBS for 10 minutes at room temperature. RA FLSs were incubated with anti-SAE1 or anti-UBA2 antibody (diluted 1:50) for 1 hour at room temperature and then incubated with FITC-conjugated secondary antibody (A-21206, Thermo Fisher Scientific). For measurement of pseudopodia organization, cells were incubated with Alexa Fluor 546 rhodamine-phalloidin (Molecular Probes, Thermo Fisher Scientific), and nuclei were visualized using 0.25 mg/mL DAPI. The coverslips were mounted on glass slides with antifade mounting medium and evaluated using fluorescence microscopy (Olympus BX63).

### Measurement of glucose uptake.

Glucose uptake was detected using a Glucose Uptake Colorimetric Assay Kit (BioVision) according to the manufacturer’s instructions. All experiments were performed in triplicate.

### Detection of lactate secretion.

Lactate levels in cell culture supernatants were measured using a Lactate Colorimetric Assay Kit (BioVision) according to the manufacturer’s instructions. All experiments were performed in triplicate.

### FLS proliferation assays.

FLS were starved for 24 hours at a density of 1 × 10^4^/well in 96-well plates in serum-free medium. After starving, FLSs were cultured under the various indicated conditions for 24 or 48 hours and then incubated with EdU (50 μM) for 8 hours. FLS proliferation was detected using a Cell-Light EdU DNA Cell Proliferation Kit (RiboBio) according to the manufacturer’s instructions.

### FLS apoptosis assays.

Cell apoptosis was performed by staining cells with FITC annexin V and PI (both from BD Biosciences) according to the manufacturer’s instructions. Briefly, FLSs were suspended in 1× binding buffer at a concentration of 1 × 10^6^ cells/mL. The cell suspension (100 μL) was then transferred to a 1.5 mL Eppendorf tube, mixed with 5 μL annexin V and 5 μL PI, and incubated for 20 minutes at room temperature in darkness. The samples were analyzed by flow cytometry within 1 hour.

### Measurement of HK, PFK, and PK activity.

For the HK activity assay, cells (1 × 10^5^) were harvested and homogenized with 200 μL ice-cold HK Assay Buffer (BioVision) for 10 minutes. The cells were centrifuged at 15,000*g* for 5 minutes, and the supernatant was assayed using a Hexokinase Colorimetric Assay Kit (BioVision). The reaction mixture was incubated at room temperature for 20–60 minutes and measured at 450 nm in a microplate reader (TECAN).

For the PFK activity assay, cells (1 × 10^5^) were harvested and homogenized with 200 μL ice-cold PFK Assay Buffer (MilliporeSigma) for 10 minutes. The cells were centrifuged at 13,000*g* for 10 minutes, and the supernatant was assayed by a Phosphofructokinase Activity Colorimetric Assay kit (MilliporeSigma). The reaction mixture was incubated at room temperature and measured at 450 nm every 5 minutes.

For the PK activity assay, cells (1 × 10^5^) were harvested and extracted with 4 volumes of the assay buffer (BioVision). The cells were centrifuged at 15,000*g* for 10 minutes to obtain a clear extract. After centrifugation, the supernatant was assayed by a Pyruvate Kinase Activity Assay Kit (BioVision). The reaction mixture was incubated for 10–20 minutes at room temperature and measured at 570 nm with a microplate reader.

### Measurement of ECAR and OCR.

The ECAR and cellular OCR were measured using the Seahorse XFe96 Extracellular Flux Analyzer (Seahorse Bioscience). Experiments were performed according to the manufacturer’s instructions. ECAR and OCR were measured using the Seahorse XF Glycolysis Stress Test Kit and Seahorse XF Cell Mito Stress Test Kit (Agilent Technologies), respectively. The probes were hydrated with 200 μL of Seahorse XF Calibrant overnight at 37°C in a non-CO_2_ incubator before the experimental run, and 4 × 10^4^ cells per well were then seeded into a Seahorse XF96 cell culture microplate for 12 hours. The cells were used for measurement of the ECAR and OCR. First, baseline measurements were obtained. Then, to measure ECAR, glucose, the oxidative phosphorylation inhibitor oligomycin, and the glycolytic inhibitor 2-DG were sequentially injected into each well at the indicated time points, and to measure OCR, oligomycin, the reversible inhibitor of oxidative phosphorylation p-trifluoromethoxy carbonyl cyanide phenylhydrazone, and the mitochondrial complex I inhibitor rotenone plus the mitochondrial complex III inhibitor antimycin A were sequentially injected. Data were analyzed by Seahorse XF96 Wave software. OCR is reported in pmol/min and ECAR in mpH/min. The results were normalized to total protein count per well.

### RNA sequencing and bioinformatics analysis.

Total RNA from each sample was quantified using a NanoDrop ND-1000 instrument, and random primers were used to generate cDNA. Total RNA (1~2 μg) was used to prepare the sequencing library in the following steps: (a) total RNA was enriched by oligo(dT) magnetic beads (rRNA was removed), and (b) the RNA sequencing library was prepared using KAPA Stranded RNA-Seq Library Prep Kit (Illumina), which incorporates dUTP into the second cDNA strand and renders the RNA sequencing library strand specific. The completed libraries were validated with an Agilent 2100 Bioanalyzer and quantified by an absolute quantification quantitative PCR method. To sequence the libraries, the barcoded libraries were mixed, denatured to single-stranded DNA in NaOH, captured on an Illumina flow cell, amplified in situ, and subsequently sequenced for 150 cycles from both ends on an Illumina HiSeq 4000 instrument.

Raw sequencing data generated from Illumina HiSeq 4000 that passed the Illumina purity filter were used for the following analysis. Trimmed reads (trimmed 5′, 3′-adapter bases) were aligned to the reference genome. Based on statistical analysis of the alignment (mapping ratio, rRNA/mitochondrial RNA content, fragment sequence bias), we determined whether the results could be used for subsequent data analysis. If so, the expression profiling, differentially expressed genes, and differentially expressed transcripts were determined. Novel genes and transcripts were also predicted. Principal components analysis, correlation analysis, hierarchical clustering, Gene Ontology, and pathway analysis scatter plots and volcano plots were created for the differentially expressed genes in R or Python software for statistical computing and graphics. The RNA sequencing data discussed in this article were deposited in the National Center for Biotechnology Information’s Gene Expression Omnibus database (GSE155766).

### Administration of GA in mice with CIA.

A total number of 16 male 7- to 8-week-old DBA/1J mice (Shanghai SLAC Laboratory Animal Co. Ltd) were injected intradermally at the base of the tail with 200 μg of bovine type II collagen (MilliporeSigma) diluted in acetic acid and emulsified at a 1:1 ratio (vol/vol) in Freund’s complete adjuvant. Twenty-one days after primary immunization, the mice were boosted by i.p. injection of bovine type II collagen emulsified at a 1:1 ratio (vol/vol) in incomplete Freund’s adjuvant. The animals were chosen randomly to be administered an i.p. injection of GA (30 mg × kg^–1^, every day, *n* = 8) or DMSO (vehicle, *n* = 8) for 21 days, which was initiated on the second day of booster immunization (day 22). Signs of arthritis were monitored every other day using a previously described scoring system (50). The arthritic score was evaluated for all 4 paws of the mice. All mice were anesthetized with 110 mg × kg^–1^ ketamine and 4.8 mg × kg^–1^ xylazine, and their hind limbs were removed and fixed in 4% paraformaldehyde. The tissue samples were decalcified in 8% formic acid and embedded in paraffin. Sections (5 μm) were stained with H&E. An inflammation score was obtained using the scoring system described previously (50).

To evaluate the effect of GA on the profiles of monokines and lymphokines in the ankle tissues of CIA mice, joints were cut into small pieces, frozen with liquid nitrogen, crushed in a mortar and pestle, and then solubilized in PBS. The cytokine levels of joint tissue extract were quantitatively determined with the Milliplex Map Mouse Cytokine kit (MilliporeSigma) according to the manufacturer’s instructions. The samples were run through FLEXMAP 3D with xPONENT software.

To determine the toxic effect of GA on CIA mice, biochemical analyses of serum samples were examined by a colorimetric enzymatic method using spectrophotometry. Serum creatinine was used as a marker of renal function. The enzymatic activities of AST and ALT were detected to evaluate changes in liver function. Histopathology of the liver and kidney was also observed by staining with H&E.

### Treatment with GA in TgTC mice.

TgTC mice were donated by Guangdong Laboratory Animals Monitoring Institute (Guangzhou, China). The TgTC mice were generated using a human TNF/β-globin recombinant gene construct, which contains a 2.8 kb fragment with the entire coding region and promoter of the human TNF-α (hTNF-α) gene, fused to a 0.77 kb fragment with the 3′ untranslated region and polyadenylation site of human β-globin replacing that of the hTNF-α gene, as previously reported (51). The fragment was then microinjected into pronuclei of FVB/J inbred strain fertilized eggs. Finally, the injected fertilized eggs were implanted into the oviduct of 8-week-old female pseudopregnant ICR mice. FVB/J mice were purchased from Beijing Vital River Laboratory Animal Technology Co., Ltd, and ICR mice were provided by Guangdong Laboratory Animals Monitoring Institute. Transgenic lineages were established by backcrossing the transgenic founder individuals to the FVB/J inbred strain. A total of 16 female 2-week-old TgTC mice were grouped randomly to be administered an i.p. injection of GA (30 mg × kg^–1^, every day, *n* = 8) or DMSO (vehicle, *n* = 8) for 6 weeks.

### Statistics.

The data are expressed as the means ± SD. Presented values were derived from at least 3 independent experiments for the in vitro experiments. We performed the experimental procedures and treatment and data analyses with blinding. To reduce baseline variability between independent experiments, we normalized the quantitative analysis of immunoblots and mRNA expression. The data were normalized as the fold change over the mean of the control. We compared 2 groups by using a 2-tailed Student’s *t* test; 3 or more groups were evaluated by 1-way ANOVA. We also used 2-way ANOVA to analyze the time × treatment interaction. A *P* value less than 0.05 was considered significant. We performed statistical analyses of the data using SPSS v13.0 software.

### Study approval.

All studies were performed according to the recommendations of the Declaration of Helsinki and approved by the Medical Ethical Committee of The First Affiliated Hospital, Sun Yat-sen University, China. All patients gave written informed consent to participate in the study. Animal handling and procedures were approved by the Animal Care and Ethics Committee of Sun Yat-sen University.

## Author contributions

CW, YX, and ML performed the majority of the experiments and analyzed and interpreted the data. JW, SX, RL, and YK performed part of the experiments. QW, YZ, XX, MS, and LL collected clinical samples and analyzed and interpreted the data. MS and JW performed the in vivo experiments. HX, CW, QW, and SGZ contributed to the study concept and design. CW, SGZ, and HX drafted the manuscript. HX contributed to the supervision of the study.

## Supplementary Material

Supplemental data

## Version 1 09/17/2020

Electronic publication
