# Supplementary material for: Increased SUMO-activating enzyme SAE1/UBA2 promotes glycolysis and pathogenic behavior of rheumatoid fibroblast-like synoviocytes
Source: JCI Insight. 2020 Sep 17;5(18):e135935. doi: 10.1172/jci.insight.135935 (PMC7526534; doi:10.1172/jci.insight.135935)
Supplement: Supplemental data [file jciinsight-5-135935-s080.pdf]

Supplementary data for figure legends, figures and Tables

Fig. S1 Effect of TNFα inhibitor etanercept (ETN) on expression of SAE1 and UBA2 in RA FLSs. RA FLSs were treated with various concentration of ETN (100, 500, 1000 μg/ml) for 48 h. (**A**) Effect of ETN on viability of RA FLSs. Cell viability was measured by MTT test. (**B** and **C**) Effect of ETN on expression of SAE1 and UBA2. Expression of SAE1 and UBA2 was measured by Western blot analysis. Data are expressed as the mean ± SD of densitometry quantification (C) from 5 independent experiments of 5 different patients. **P*<0.05 versus control (CTRL) by 1-way ANOVA

Fig. S2 Expression of SAE1 and UBA2 in synovial T cells (CD3+) and macrophages (CD68+) from patients with RA. The expression of CD3, CD68, SAE1, UBA2 and nuclei was evaluated by immunofluorescence staining using fluorescence microscopy, and representative images from three independent experiments are shown. Original magnification, 400×

Fig. S3 Efficiency of SAE1/UBA2 knockdown. RA FLSs were transfected with SAE1 or UBA2 siRNA 1–3 for 48 h and subjected to RT-qPCR analysis (A) and Western blotting (B). Data shown are the mean ± SD from 3 independent experiments. **P* < 0.05 versus control siRNA (siC), by 1-way ANOVA

Fig. S4 Effect of SAE1/UBA2 knockdown on migration, invasion and the expression of proinflammatory cytokines in HC FLSs. HC FLSs were transfected with SAE1 or UBA2 siRNAs or siC for 48 h. (**A**) Chemotactic migration of HC FLSs was evaluated using a Transwell assay. (**B**) *In vitro* invasion was determined using inserts coated with Matrigel Basement Membrane Matrix. The relative invasion rate was calculated by counting invaded cells and normalizing to the siC-treated cells. Representative images (left panel) are shown (original magnification, 100×). Data (right panel) are expressed as the mean ± SD of 3 independent experiments from 3 different HC subjects. (**C**) Effect of SAE1/UBA2 suppression on the proliferation of HC FLSs. An EdU incorporation assay was used to measure cell proliferation. Representative images show proliferation of RA FLSs labeled with EdU (red) and nuclei stained with Hoechst 33342 (blue) (original magnification, 100×). Graphs indicate the mean ± SD of 4 independent experiments involving 4 different HC subjects. (**D**) Effect of SAE1 or

UBA2 inhibition on apoptosis of RA FLSs. The cellular apoptosis rate was evaluated by annexin V and PI staining and measured by flow cytometry. Representative flow plots are shown. The apoptosis represents the mean ± SD percentage of 3 independent experiments of 3 different HC subjects. (**E**) Effect of SAE1/UBA2 inhibition on TNF-α-induced expression of IL-1β, IL-6 and IL-8. Cytokine expression was evaluated by RT-qPCR. Ct values were normalized to β-actin values. Data are presented as the mean ± SD from 4 independent experiments of 4 different HC subjects. *$P < 0.05$ versus siC, by 1-way ANOVA

Fig. S5 Effect of SAE1/UBA2-mediated SUMOylation inhibition on HK and PFK activity. RA FLSs were transfected with siRNA-1 and siRNA-3 for SAE1 (siSAE1-1, siSAE1-3) or UBA2 (siUBA2-1, siUBA2-3) or siC for 48 h or pretreated with GA (150 μM) for 24 h and then stimulated with or without TNF-α (10 ng/ml) for 24 h. Enzymatic activity was detected by the indicated kits. Data are expressed as the mean ± SD from 4 independent experiments. ***$P<0.05$ versus siC or DMSO, by 1-way ANOVA

Fig. S6 Effect of GA on TNF-α-induced translocation of PKM2 in RA FLSs. RA FLSs were pretreated with GA (150 μM) for 24 h and then stimulated with or without TNF-α (10 ng/ml) for 12 h. For cellular IF staining, PKM2 (green) and nuclei (blue) were evaluated using fluorescence microscopy, and representative images from three independent experiments are shown. Original magnification, 400×.

Fig. S7 Effect of SKN on TNF-α-induced translocation of PKM2 in RA FLSs. RA FLSs were pretreated with SKN (2 μM) for 24 h and then stimulated with or without TNF-α (10 ng/ml) for 12 h. For cellular IF staining, PKM2 (green) and nuclei (blue) were evaluated using fluorescence microscopy, and representative images from three independent experiments are shown. Original magnification, 400×.

Fig. S8 Measurement of SUMO-1-PKM2 interactions in RA FLSs. Immunoprecipitations were performed as described in the *Materials and Methods*. The precipitates were separated by SDS–PAGE, and Western blotting was performed using anti-PKM2 (right, upper panel) and anti-SUMO-1 antibodies (right, lower panel). Input controls are shown as indicated (left panel).

Fig. S9 Effect of GA treatment on SUMO-1-PKM2 interactions. Cells were treated with GA for 24 h. The immunoprecipitates were probed with anti-PKM2 (upper panels) or anti-SUMO-1 (lower panels) antibody. Immunoprecipitations were also performed with IgG as a negative control. Input controls are shown as indicated (left panel).

Fig. S10 Expression of phosphorylated PKM2 (p-PKM2) in FLSs and synovial tissue from RA patients and healthy control (HC) subjects. A, Expression of p-PKM2 in FLSs was measured by Western blot analysis. Data are expressed as the mean ± SD of densitometry quantification (right panel) from 5 independent experiments of 5 different patients. *P<0.05 versus HC, by Student's *t* test. **B**, The expression of p-PKM2 in synovial tissue from RA patients and HC subjects was detected by immunohistochemical staining. A representative image was shown from 5 RA patients or 5 HC subjects. Original magnification, 400×

Fig. S11 Efficiency of p-PKM2 knockdown. RA FLSs were transfected with PKM2 siRNA 1-3 for 48 h and subjected to RT-qPCR analysis (**A**) and Western blot (**B**). A representative blot of at least 3 independent experiments is shown. The data shown are the mean ± SD from at least 3 independent experiments. *P < 0.05 versus siC, by 1-way ANOVA

Fig. S12 Effect of PKM2 knockdown on migration, invasion and the expression of IL-1β, IL-6 and IL-8 in RA FLSs. RA FLSs were transfected with siRNA-2 and siRNA-3 for PKM2 (siPKM2-2, siPKM2-3) or siC. **A** and **B**, Effect of PKM2 knockdown on migration and invasion. Migration (**A**) and invasion (**B**) were measured with a Boyden chamber. An invasion assay was performed using inserts coated with a Matrigel basement membrane matrix. The migrated or invaded FLSs were stained violet using a Diff-Quick kit (left panel, original magnification, 100×). The migration or invasion index represents the number of migrated or invaded cells normalized to siC-treated cells. Data show the mean ± SD for samples from 4 different RA patients. **C**, Effect of PKM2 knockdown on the expression of IL-1β, IL-6 and IL-8. Cytokine expression was measured by RT-qPCR analysis. Data show the mean ± SD for samples from 5 different RA patients. *P < 0.05, versus siC; $^{\#}P$ <0.05 versus TNF-α + siC, by 1-way

ANOVA

Fig. S13 Effect of PKM2 knockdown on lactate secretion, glucose uptake and the expression of LDHA, PDK1, and GLUT1. RA FLSs were transfected with siRNA-2 and siRNA-3 for PKM2 (siPKM2 -2, siPKM2 -3) or siC, and then stimulated with or without TNF-α (10 ng/ml) for 24 h. **A**, Effect of PKM2 knockdown on lactate secretion. Lactate levels in the supernatants of cultured RA FLSs were detected using a Lactate Colorimetric Assay Kit. **B**, Effect of PKM2 knockdown on glucose uptake. Glucose uptake was detected using a colorimetric glucose uptake assay kit. **C-E**, Effect of PKM2 knockdown on the expression of LDHA (**C**), PDK1 (**D**) and GLUT1 (**E**). The expression of LDHA, PDK1 and GLUT1 was measured by RT-qPCR. The data are representative of at least 3 independent experiments (mean ± SD). $^{*}P < 0.05$, versus siC; $^{\#}P < 0.05$ versus TNF-α + siC, by 1-way ANOVA

Fig. S14 Effect of shikonin (SKN) treatment on the phosphorylation of PKM2. The expression of p-PKM2 was measured by Western blot analysis. **A,** RA FLSs were treated with SKN (2 μM) for various time points (6, 12, 24, 48 h). **B**, RA FLSs were pretreated with SKN for 24 h and then stimulated with or without TNFα (10 ng/ml) for 30 min. Data are expressed as the mean ± SD of densitometry quantification (lower panels) from 3 independent experiments of 3 different patients. $^{*}P < 0.05$ versus DMSO, $^{\#}P<0.05$ versus TNF-α + DMSO, by 1-way ANOVA

Fig. S15 Effect of shikonin (SKN) on migration, invasion and the expression of IL-1β, IL-6 and IL-8 by RA FLSs. RA FLSs were pretreated with SKN for 24 h and then stimulated with or without TNF-α (10 ng/ml) for the indicated times. **A** and **B**, Effect of SKN treatment on migration and invasion. Migration (**A**) and invasion (**B**) were measured with a Boyden chamber. An invasion assay was performed using inserts coated with a Matrigel basement membrane matrix. The migrated or invaded FLSs were stained violet using a Diff-Quick kit (left panel, original magnification, 100×). The migration or invasion index represents the number of migrated or invaded cells normalized to DMSO-treated cells. **C**, Effect of SKN treatment on the expression of IL-1β, IL-6 and IL-8. Cytokine expression was measured by RT-qPCR analysis. Data show the mean ± SD for samples from at least 3 different RA patients.$^{*}P < 0.05$

versus DMSO, $^{\#}$*P*<0.05 versus TNF-α + DMSO, by Student's *t* test (for panel A and B) or 1-way ANOVA (for panel C)

Fig. S16 Effect of shikonin (SKN) on lactate secretion, glucose uptake and the expression of LDHA, PDK1, and GLUT1. RA FLSs were pretreated with SKN for 24 h and then stimulated with or without TNF-α (10 ng/ml) for the indicated times. **A**, Effect of SKN treatment on lactate secretion. Lactate levels in the supernatants of cultured RA FLSs were detected using a Lactate Colorimetric Assay Kit. **B**, Effect of SKN treatment on glucose uptake. Glucose uptake was detected using a colorimetric glucose uptake assay kit. **C-E**, Effect of SKN treatment on the expression of LDHA (**C**), PDK1 (**D**) and GLUT1 (**E**). The expression of LDHA, PDK1 and GLUT1 was measured by RT-qPCR. The data are representative of 4 independent experiments (mean ± SD). **P* < 0.05 versus DMSO, $^{\#}$*P*<0.05 versus TNF-α + DMSO, by 1-way ANOVA

Fig. S17 Effect of the K336R mutation on TNF-α-induced expression of STAT5A in RA FLSs. Data (B) are expressed as the mean ± SD of densitometry quantification of Western blot results from 3 independent experiments. **P*<0.05 versus WT; $^{\#}$*P*<0.05 vs. TNF-α +WT, by 1-way ANOVA

Fig. S18 Silencing efficiency of STAT5A siRNA. RA FLSs were transfected with STAT5A siRNA 1–3 for 48 h and subjected to RT-qPCR analysis (A) and Western blot (B). A representative blot of 3 independent experiments is shown. The data shown are the mean ± SD from 3 independent experiments. **P* < 0.05 versus control siRNA (siC) by 1-way ANOVA

Fig. S19 Effect of the SAE1/UBA2 inhibitor GA on frequencies of FLSs, T cells (CD3+) and macrophages (F4/80+) in synovial tissue from mice with CIA. DBA/1J male mice were immunized with bovine type II collagen in CFA and administered a booster injection 21 d later to induce CIA. On the 22nd day, the mice were injected i.p. with GA (30 mg/kg/d) or DMSO (as a model control) daily for 21 d. The expression of FLS (vimentin), F4/80, CD3 and nuclei was evaluated by immunofluorescence staining using fluorescence microscopy. Representative images (left panel) and quantification of the percentage of synovial vimentin-, F4/80- and CD3-positive cells (right panel) of DMSO-treated (n = 8) and GA-treated (n =8) mice (original magnification, 400×). **P* <

0.05 versus DMSO, by Student's *t* test

Fig. S20 Expression of SAE1 and UBA2 in synovial T cells (CD3+) and macrophages (F4/80+) from mice with CIA. DBA/1J male mice were immunized with bovine type II collagen in CFA and administered a booster injection 21 d later to induce CIA. On the 22nd day, the mice were injected i.p. with GA (30 mg/kg/d) or DMSO (as a model control) daily for 21 d. The expression of synovial CD3, F4/80, SAE1, UBA2 and nuclei was evaluated by immunofluorescence staining using fluorescence microscopy. Representative images (left panel) and quantification of the percentage of CD3+SAE1+, F4/80+SAE1+, CD3+UBA2+and F4/80+ UBA2+ cells (right panel) of DMSO-treated (n = 8) and GA-treated (n =8) mice (original magnification, 400×). *$P <$ 0.05 versus DMSO, by Student's *t* test

Fig.S1

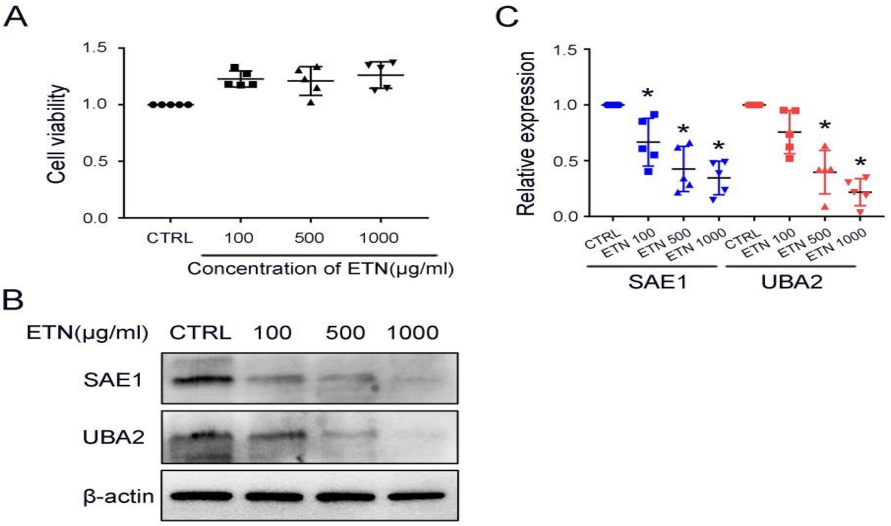
A
Cell viability
1.5
1.0
0.5
0.0
CTRL
100
500
1000
Concentration of ETN(μg/ml)
B
ETN(μg/ml) CTRL 100 500 1000
SAE1
UBA2
β-actin
C
Relative expression
1.5
1.0
0.5
0.0
CTRL
ETN 100
ETN 500
ETN 1000
*
SAE1
UBA2

Fig.S2

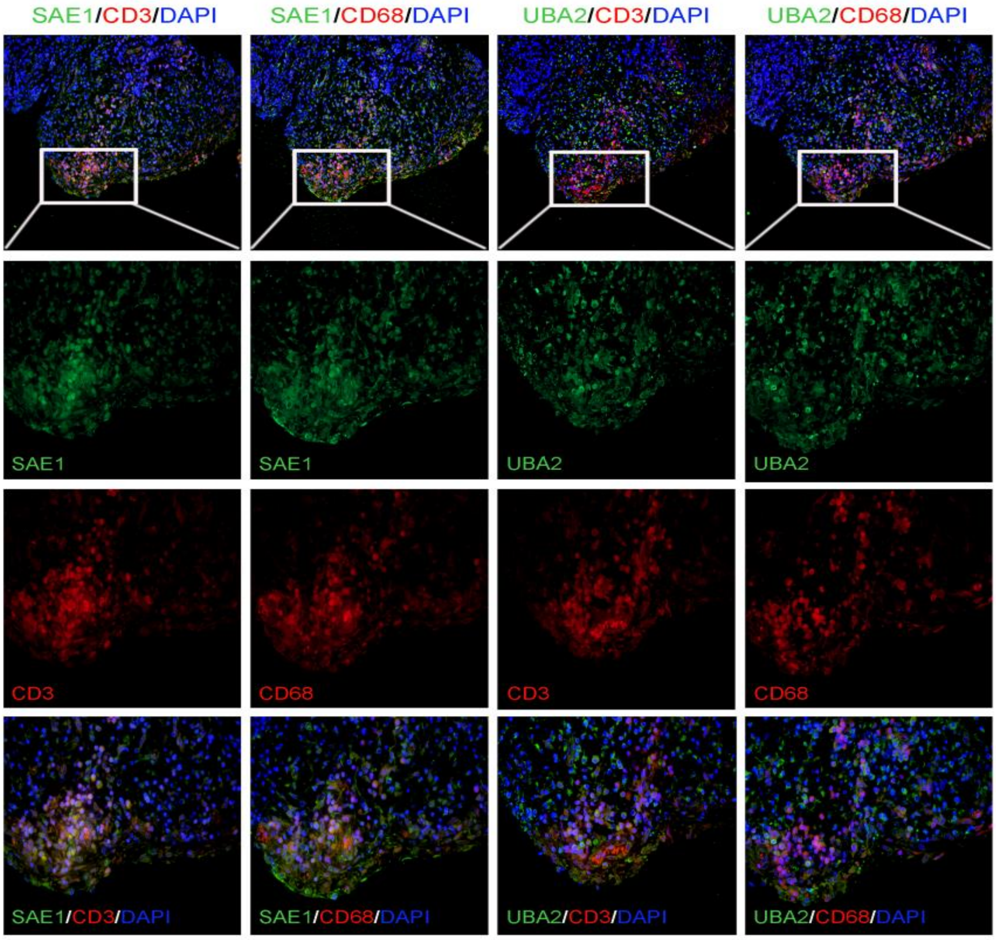
SAE1/CD3/DAPI
SAE1/CD68/DAPI
UBA2/CD3/DAPI
UBA2/CD68/DAPI
SAE1
SAE1
UBA2
UBA2
CD3
CD68
CD3
CD68
SAE1/CD3/DAPI
SAE1/CD68/DAPI
UBA2/CD3/DAPI
UBA2/CD68/DAPI

Fig.S3

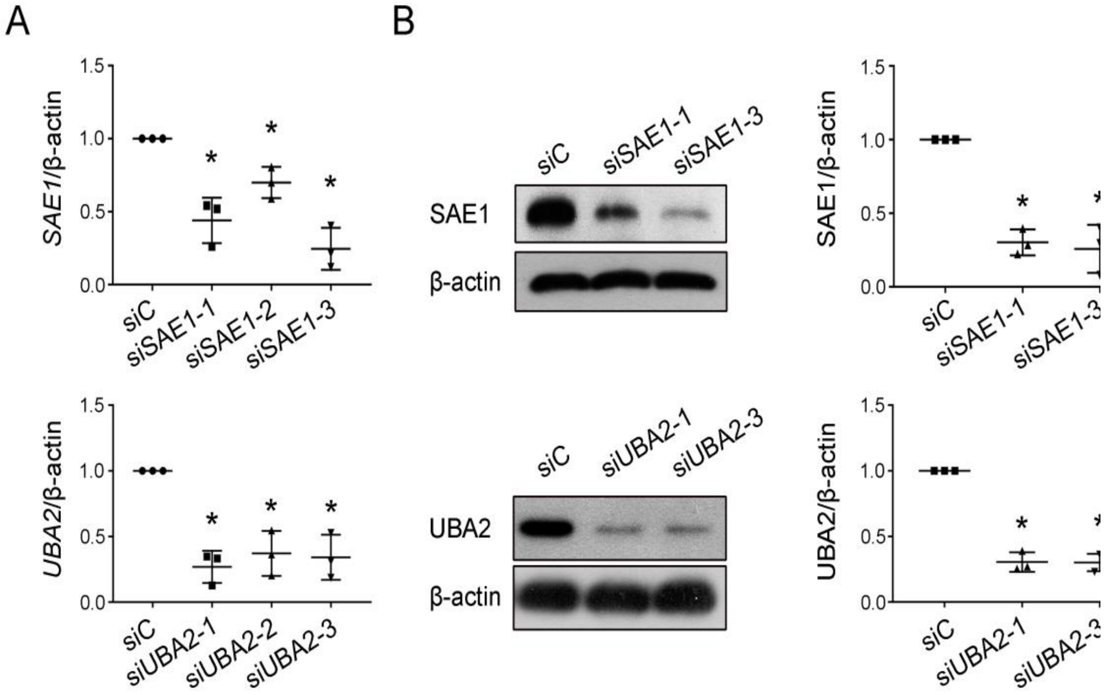
A
B
1.5
1.0
0.5
0.0
SAE1/β-actin
siC
siSAE1-1
siSAE1-2
siSAE1-3
UBA2/β-actin
siUBA2-1
siUBA2-2
siUBA2-3
siSAE1-3
SAE1
β-actin
UBA2

Fig.S4

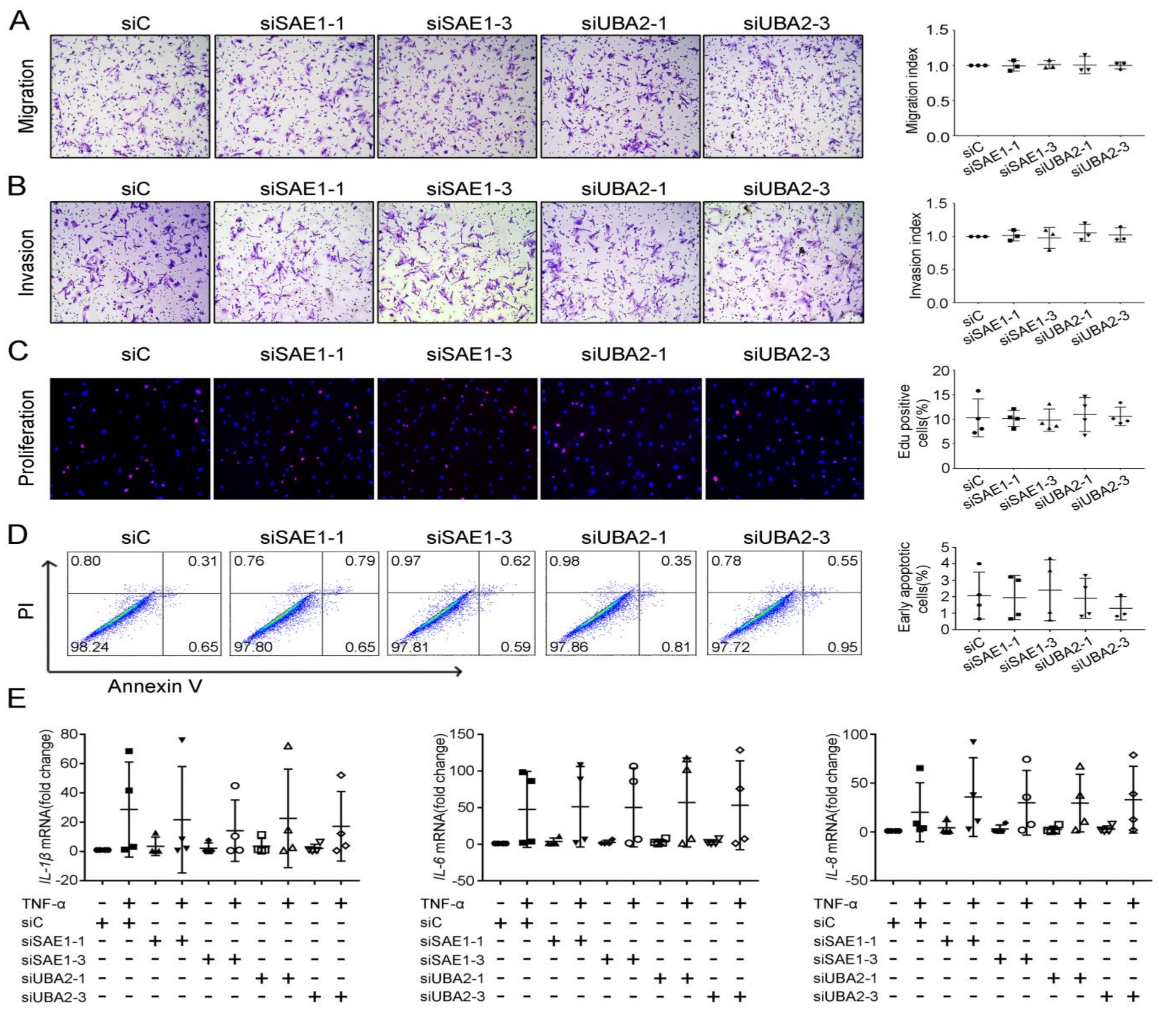
A
B
C
D
E
siC
siSAE1-1
siSAE1-3
siUBA2-1
siUBA2-3
Migration
Invasion
Proliferation
Migration index
Invasion index
Edu positive cells(%)
Early apoptotic cells(%)
PI
Annexin V
0.80 0.31 98.24 0.65
0.76 0.79 97.80 0.65
0.97 0.62 97.81 0.59
0.98 0.35 97.86 0.81
0.78 0.55 97.72 0.95
IL-1β mRNA(fold change)
IL-6 mRNA(fold change)
IL-8 mRNA(fold change)
TNF-α
siC
siSAE1-1
siSAE1-3
siUBA2-1
siUBA2-3

Fig.S5

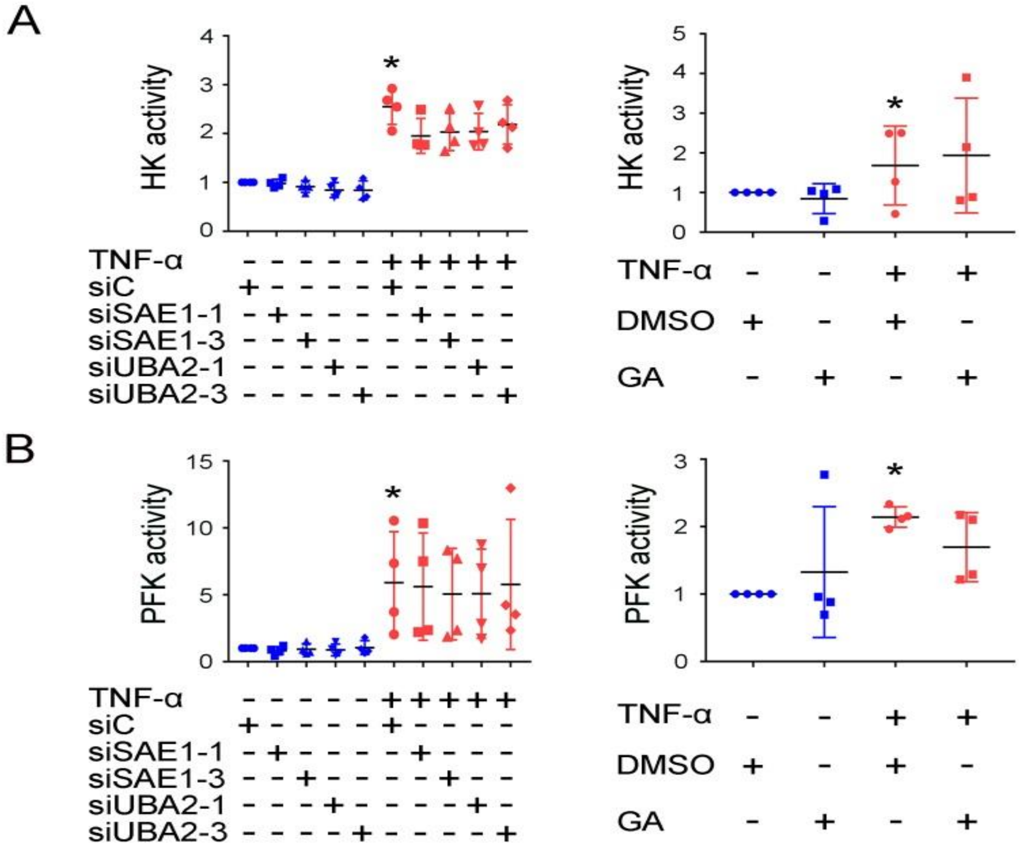
A
HK activity
TNF-α
siC
siSAE1-1
siSAE1-3
siUBA2-1
siUBA2-3
HK activity
TNF-α
DMSO
GA
B
PFK activity
TNF-α
siC
siSAE1-1
siSAE1-3
siUBA2-1
siUBA2-3
PFK activity
TNF-α
DMSO
GA

Fig.S6

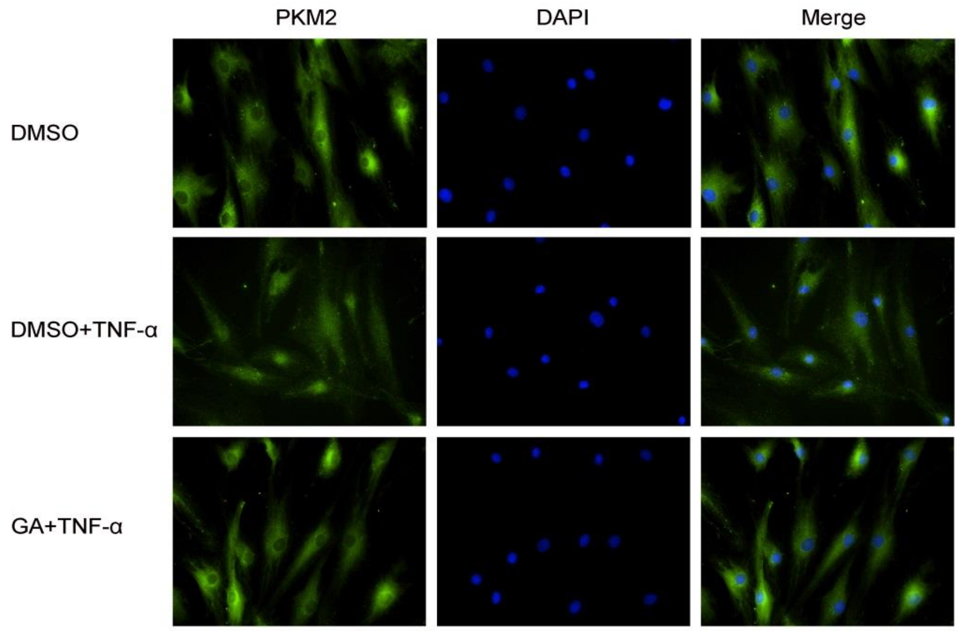
PKM2
DAPI
Merge
DMSO
DMSO+TNF-α
GA+TNF-α

Fig.S7

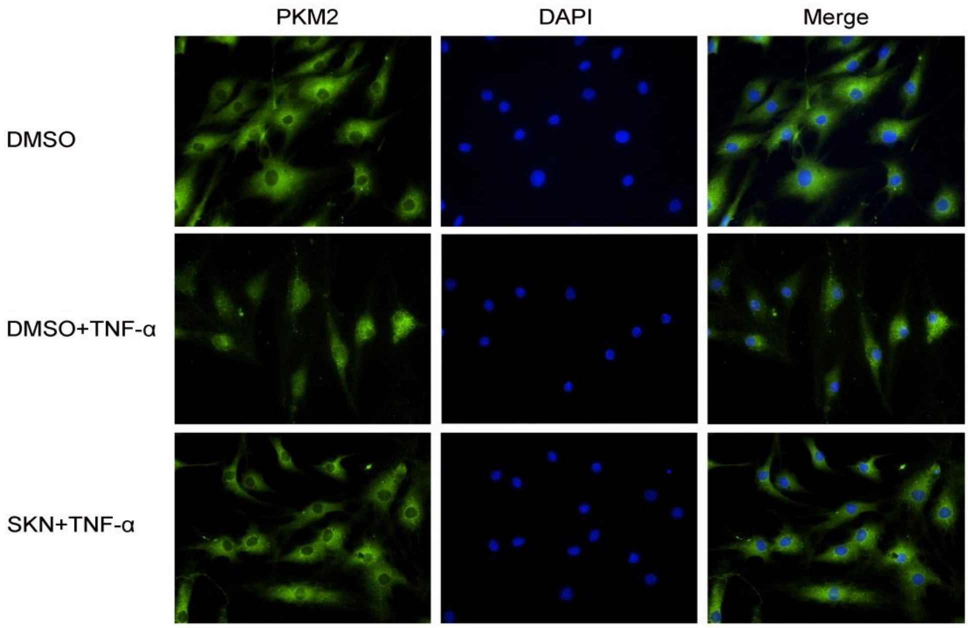
PKM2
DAPI
Merge
DMSO
DMSO+TNF-α
SKN+TNF-α

Fig.S8

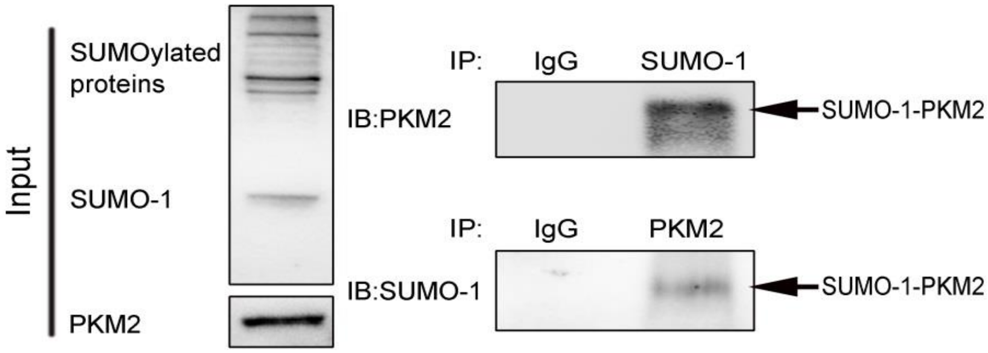
SUMOylated proteins
Input
SUMO-1
PKM2
IB:PKM2
IB:SUMO-1
IP:
IgG
SUMO-1
SUMO-1-PKM2
IP:
IgG
PKM2
SUMO-1-PKM2

Fig.S9

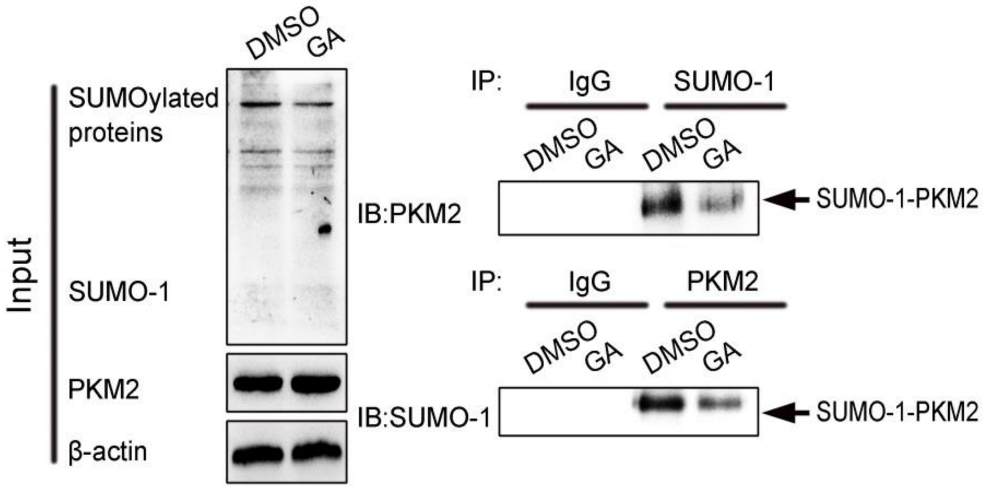
DMSO
GA
SUMOylated proteins
Input
SUMO-1
PKM2
β-actin
IB:PKM2
IB:SUMO-1
IP:
IgG
SUMO-1
DMSO
GA
DMSO
GA
SUMO-1-PKM2
IP:
IgG
PKM2
DMSO
GA
DMSO
GA
SUMO-1-PKM2

Fig.S10

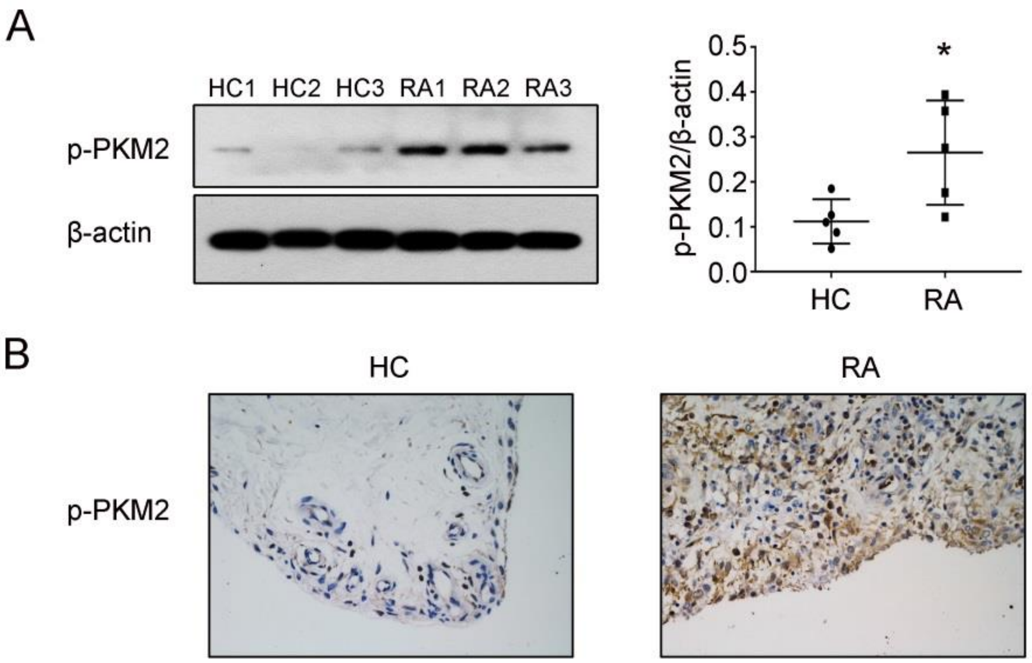

Fig.S11

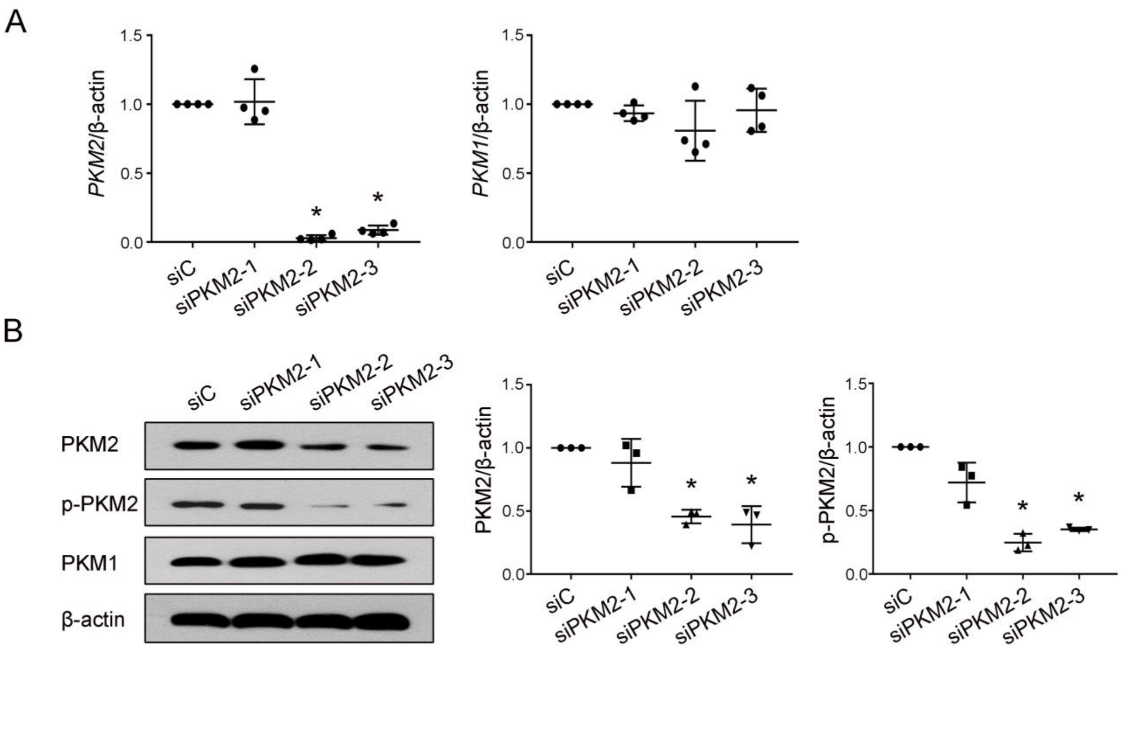

Fig.S12

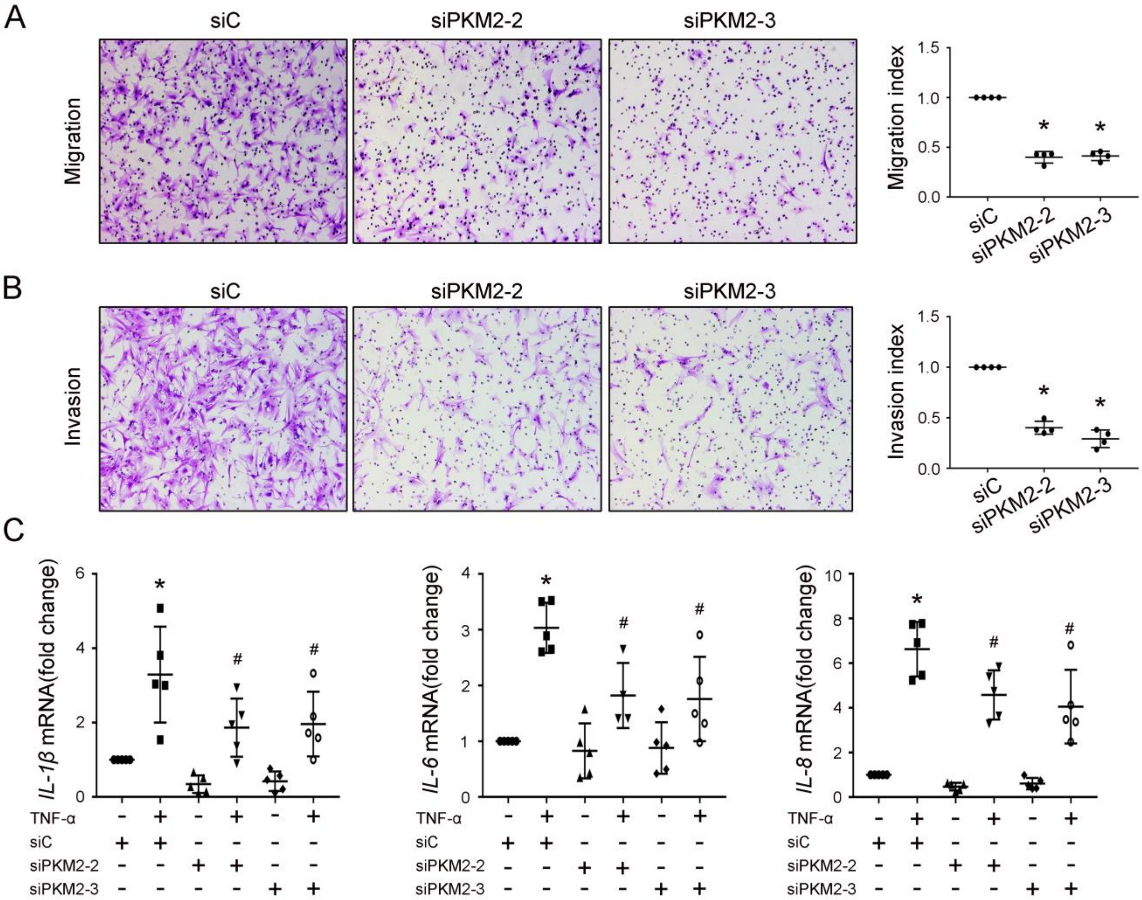
A
siC
siPKM2-2
siPKM2-3
Migration
Migration index
1.5
1.0
0.5
0.0
siC
siPKM2-2
siPKM2-3
B
siC
siPKM2-2
siPKM2-3
Invasion
Invasion index
1.5
1.0
0.5
0.0
siC
siPKM2-2
siPKM2-3
C
IL-1β mRNA(fold change)
IL-6 mRNA(fold change)
IL-8 mRNA(fold change)
TNF-α
siC
siPKM2-2
siPKM2-3

Fig.S13

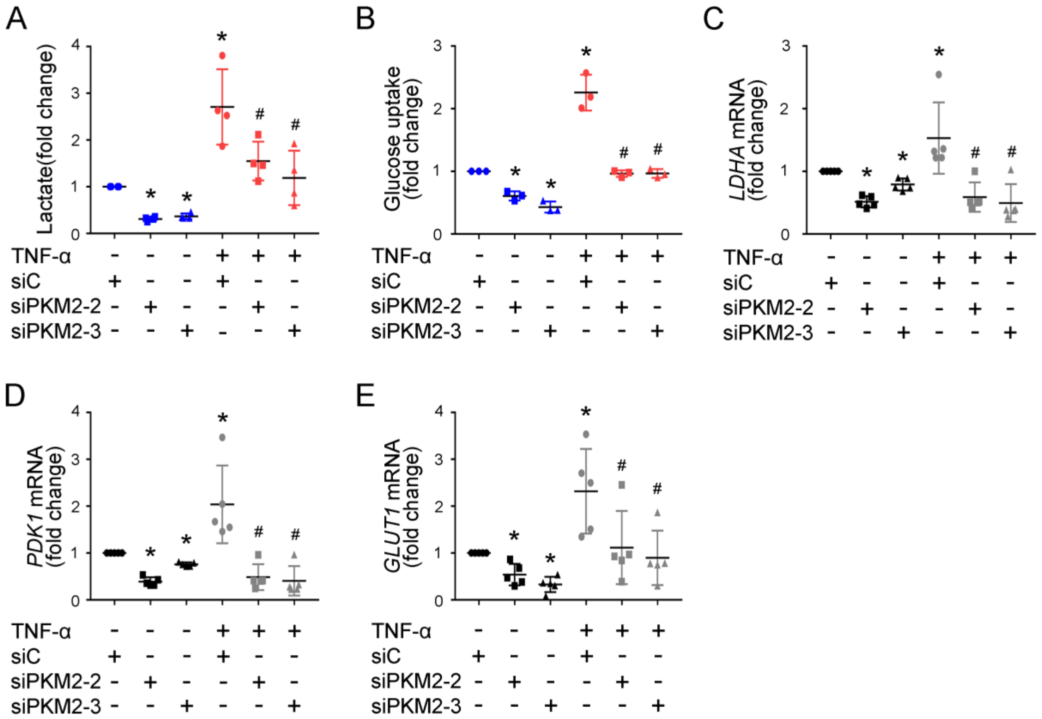

Fig.S14

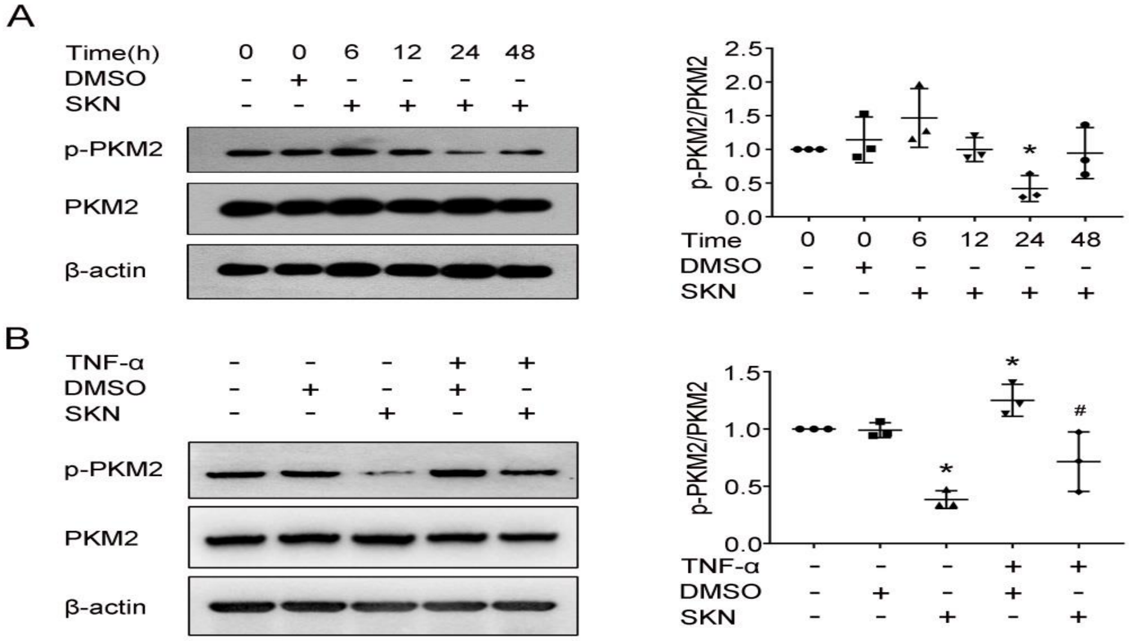

Fig.S15

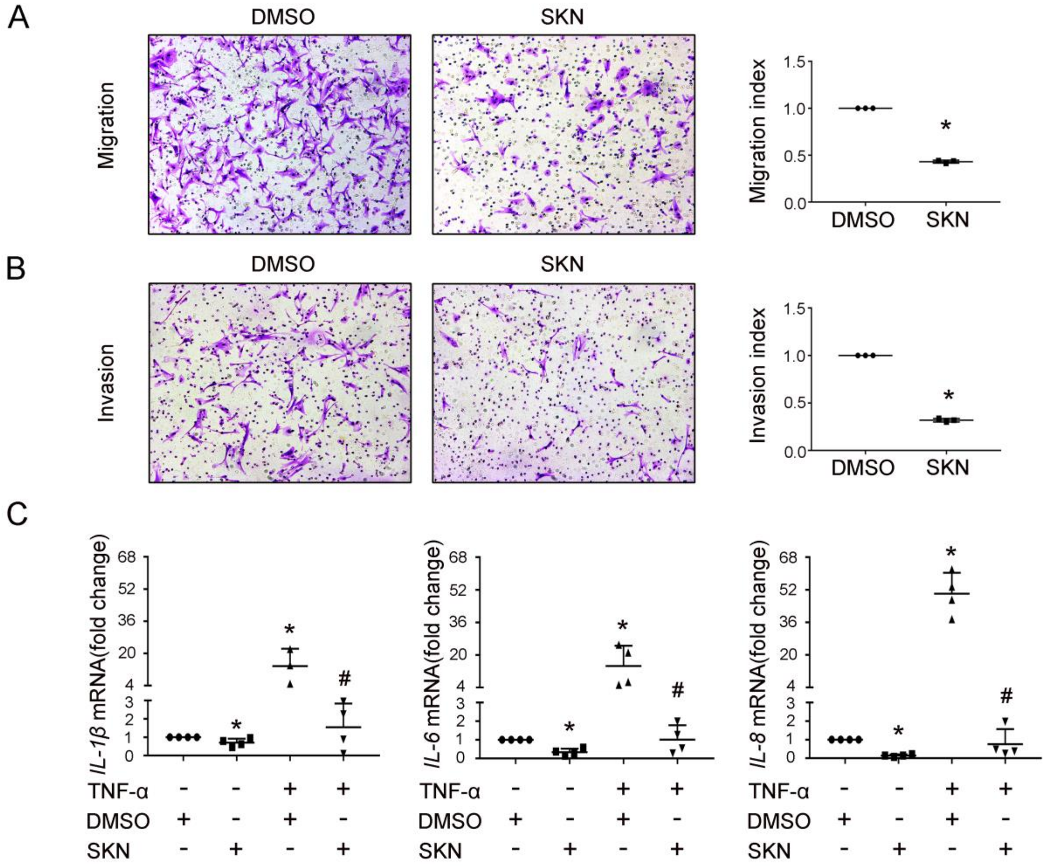
A
DMSO
SKN
Migration
Migration index
DMSO
SKN
B
DMSO
SKN
Invasion
Invasion index
DMSO
SKN
C
IL-1β mRNA(fold change)
IL-6 mRNA(fold change)
IL-8 mRNA(fold change)
TNF-α
DMSO
SKN

Fig.S16

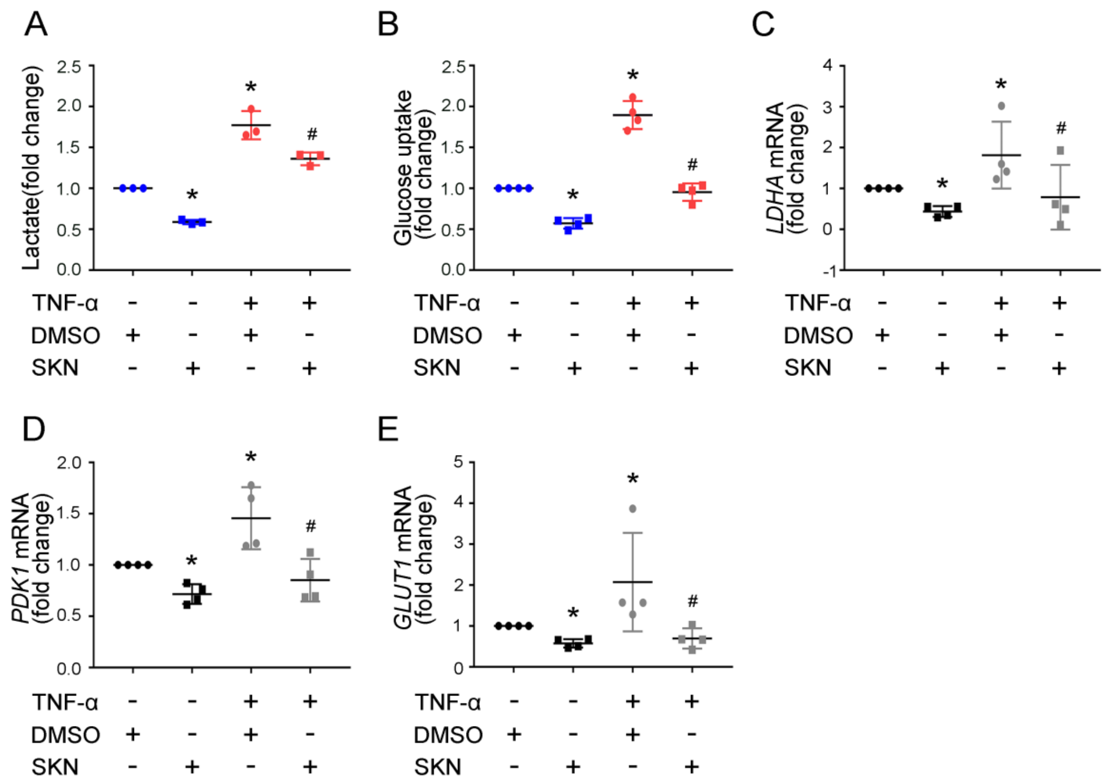

Fig.S17

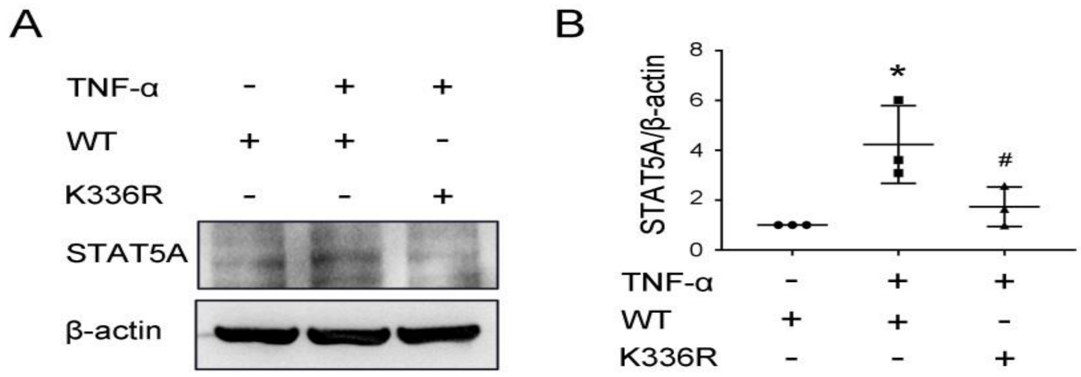

Fig.S18

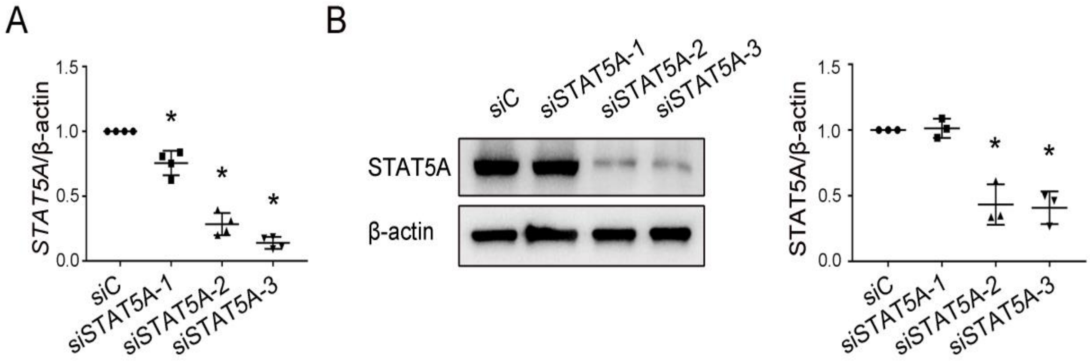

Fig.S19

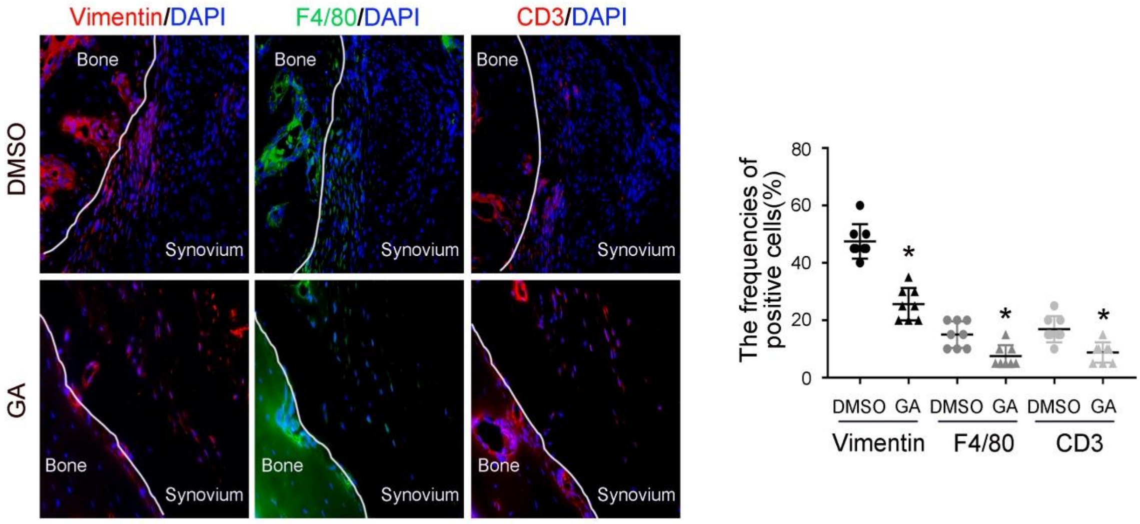

Fig.S20

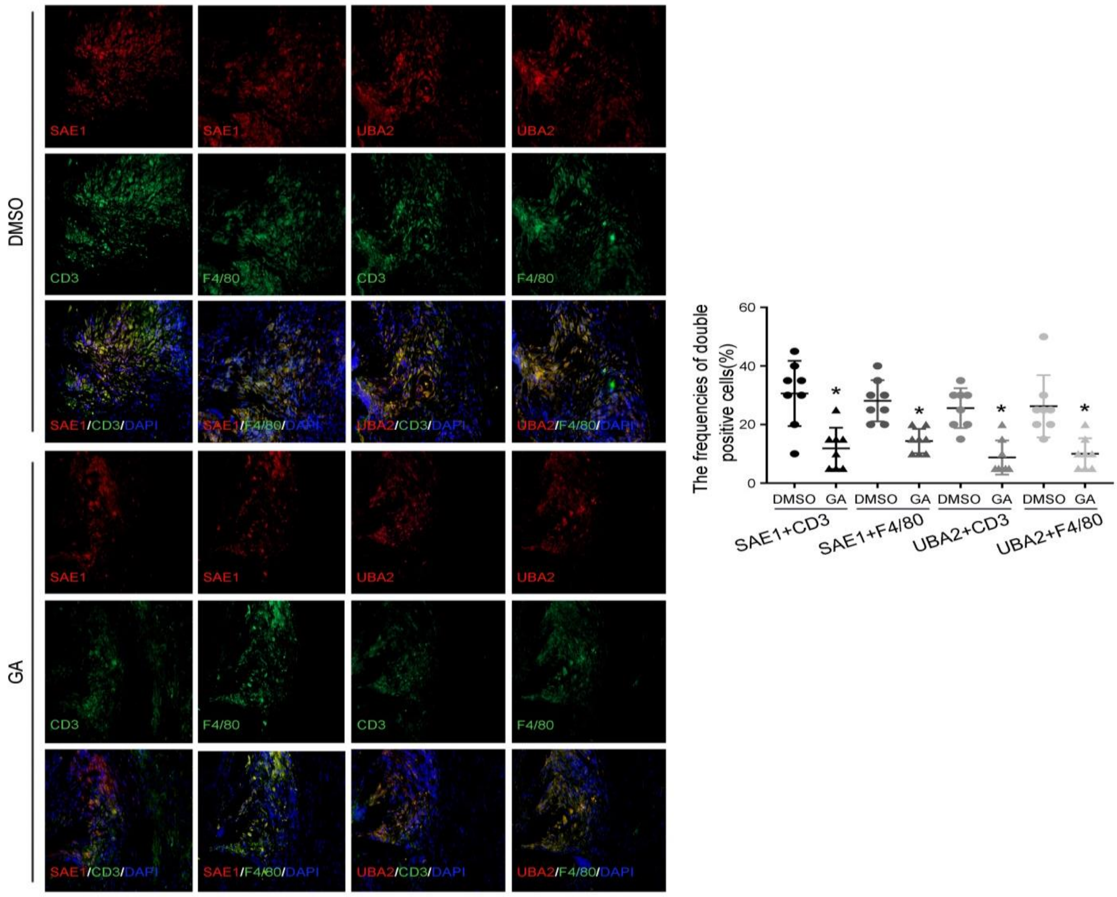

DMSO
GA
SAE1
UBA2
CD3
F4/80
SAE1/CD3/DAPI
SAE1/F4/80/DAPI
UBA2/CD3/DAPI
UBA2/F4/80/DAPI
The frequencies of double positive cells(%)
60
40
20
0
DMSO
GA
SAE1+CD3
SAE1+F4/80
UBA2+CD3
UBA2+F4/80
*

Tables

Table S1. Demographic and clinical features of patients with active RA.

Age, yrs (mean±SD)	54.8±7.5
Female, n (%)	20（83.3）
Male, n (%)	4 (16.7)
Disease duration, yrs (mean±SD)	6 (7.8)
Rheumatoid factor-positive, n (%)	20（83.3）
Anti-CCP-positive, n (%)	16 (66.7)
DAS28 (CRP) (mean±SD)	5.4 ± 1.5
Previous medications, n (%)	
Prednisone (<10mg/d)	12 (50)
Methotrexate	10 (41.6)
Leflunomide	4(16.7)
Sulfasalazine	3 (12.5)
Hydroxychloroquine	6 (25)

Table S2 1 Sequences of primers and siRNA

		RT-qPCR primers
AHRR	Forward	AGGTGCCAGATTGAGTGGTG
	Reverse	TTGCCGTTGAGGAAGGGATG
ARHGAP11A	Forward	ATATTGGGCGTGTACCAGATTTT
	Reverse	CAATGTACGCTTAGCATTTGGTG
β-actin	Forward	TCAAGATCATTGCTCCTCCTGAG
	Reverse	ACATCTGCTGGAAGGTGGACA
CCL2	Forward	CAGCCAGATGCAATCAATGCC
	Reverse	TGGAATCCTGAACCCACTTCT
GLUT1	Forward	ATACACCACCTCACTCCTGTT
	Reverse	CCATCCAAACCTCCTACCCT
HK1	Forward	TGGCTCATTTCCACCTCACC
	Reverse	GCATTGTTGTGCGTCTGCTT
HK2	Forward	TGCCACCAGACTAAACTAGACG
	Reverse	CCCGTGCCCACAATGAGAC
HK3	Forward	TGAGGGGCATAGGGTGGAG
	Reverse	GTGGGCAGCAAAGTCAAAGAG
HK4	Forward	CAGGTCACAGAAGGGAGAGG
	Reverse	CTGGTTTGGGGTTTGAGGTT
IL-1β	Forward	ATGATGGCTTATTACAGTGGCAA
	Reverse	GTCGGAGATTCGTAGCTGGA
IL-6	Forward	ACTCACCTCTTCAGAACGAATTG
	Reverse	CCATCTTTGGAAGGTTCAGGTTG
IL-8	Forward	ACTGAGAGTGATTGAGAGTGGAC
	Reverse	AACCCTCTGCACCCAGTTTTC
LDHA	Forward	GTGTGCCTGTATGGAGTGGA
	Reverse	AGGATGTGTAGCCTTTGAGTTTG
PDK1	Forward	CCAAGACCTCGTGTTGAGACC
	Reverse	AATACAGCTTCAGGTCTCCTTGG
PFKL	Forward	CTCGTGGAGGGAGGTGAGAA
	Reverse	TGACGCACAGGTTGGTGATG
PFKM	Forward	GGAGATGCCCAAGGTATGAATG
	Reverse	CACCCCCAATGACACAGAGA
PFKP	Forward	CGCCTACCTCAACGTGGTG
	Reverse	ACCTCCAGAACGAAGGTCCTC
PKL	Forward	CAGACCCACAGAGAGGGAGA
	Reverse	TCAATGTCCAGTAGGCAGAGG
PKM1	Forward	CGAGCCTCAAGTCACTCCAC
	Reverse	GTGAGCAGACCTGCCAGACT
PKM2	Forward	ATTATTTGAGGAACTCCGCCGCCT

	Reverse	ATTCCGGGTCACAGCAATGATGG
PKR	Forward	GGATGGATGCTGGGGAGAAG
	Reverse	GCTCTTGGTTTGTGTGTGGG
SAE1	Forward	GTGAAAGGACTGACCATGCTG
	Reverse	CCCAGTACGAATCAAGAACTGAG
STAT5A	Forward	GCAGAGTCCGTGACAGAGG
	Reverse	CCACAGGTAGGGACAGAGTCT
SUMO-1	Forward	TGACCAGGAGGCAAAACCTTC
	Reverse	AATTCATTGGAACACCCTGTCTT
THAP6	Forward	AGGACTGACATTTCACGTATTCC
	Reverse	CCGGCTGCATTCACATCAA
UBA2	Forward	GTTTCTCCCACATCGACCTGAT
	Reverse	GGCAACCTGTGCCTTTGATCTT

		**siRNA**
PKM2	siRNA-1	CATCTACCACTTGCAATTA
	siRNA-2	CCATAATCGTCCTCACCAA
	siRNA-3	CTACCACTTGCAATTATTTGA
SAE1	siRNA-1	AGACAACGATGGTCAAAAA
	siRNA-2	GTGCTTCTTGTCGGCTTGA
	siRNA-3	AGCGAGCTCAGAATCTCAA
STAT5A	siRNA-1	TCCGGCACATTCTGTACAA
	siRNA-2	GAACTCTTACGCCGACCAA
	siRNA-3	TCTTGTTGCGCTTTAGTGA
SUMO-1	siRNA-1	CTGGGAATGGAGGAAGAAG
	siRNA-2	CAATGAATTCACTCAGGTT
	siRNA-3	CAAGAAACTCAAAGAATCA
UBA2	siRNA-1	CCGACAGTTTATACTGGTT
	siRNA-2	CAAAGAGGTCACGTATAGA
	siRNA-3	CATCGAGACTTTGAGAGTT

Table S3 Effect of GA on body weight, blood glucose, liver and kidney functions of CIA mice (n=8)

Group	Weight(g)	Blood glucose(mmol/L)	ALT(IU/L)	AST(IU/L)	Scr(μmol/L)
DMSO	24.86±2.15	10.18±3.23	13.79±4.57	13.11±3.19	26.24±9.48
GA	25.31±1.62	8.73±2.95	10.85±2.47	13.13±3.82	24.62±4.69

ALT-alanine aminotransferase, AST-aspartate aminotransferase, Scr-serum creatinine
